# Drug–Phospholipid Co-Amorphous Formulations: The Role of Preparation Methods and Phospholipid Selection

**DOI:** 10.3390/pharmaceutics16121602

**Published:** 2024-12-16

**Authors:** Keyoomars Khorami, Sam Darestani Farahani, Anette Müllertz, Thomas Rades

**Affiliations:** Department of Pharmacy, Faculty of Health and Medical Science, University of Copenhagen, Universitetsparken 2, DK-2100 Copenhagen, Denmark; keyoomars.khorami@sund.ku.dk (K.K.); wpr774@alumni.ku.dk (S.D.F.); thomas.rades@sund.ku.dk (T.R.)

**Keywords:** amorphization, ball milling, co-amorphous systems, drug stability, dissolution enhancement, phospholipids, poorly water-soluble drugs, quench cooling, solid state characterization, solvent evaporation

## Abstract

**Background/Objectives**: This study aims to broaden the knowledge on co-amorphous phospholipid systems (CAPSs) by exploring the formation of CAPSs with a broader range of poorly water-soluble drugs, celecoxib (CCX), furosemide (FUR), nilotinib (NIL), and ritonavir (RIT), combined with amphiphilic phospholipids (PLs), including soybean phosphatidylcholine (SPC), hydrogenated phosphatidylcholine (HPC), and mono-acyl phosphatidylcholine (MAPC). **Methods**: The CAPSs were initially prepared at equimolar drug-to-phospholipid (PL) ratios by mechano-chemical activation-based, melt-based, and solvent-based preparation methods, i.e., ball milling (BM), quench cooling (QC), and solvent evaporation (SE), respectively. The solid state of the product was characterized by X-ray powder diffraction (XRPD), polarized light microscopy (PLM), and differential scanning calorimetry (DSC). The long-term physical stability of the CAPSs was investigated at room temperature under dry conditions (0% RH) and at 75% RH. The dissolution behavior of the CCX CAPS and RIT CAPS was studied. **Results**: Our findings indicate that SE consistently prepared CAPSs for CCX-PLs, FUR-PLs, and RIT-PLs, whereas the QC method could only form CAPSs for RIT-PLs, CCX-SPC, and CCX-MAPC. In contrast, the BM method failed to produce CAPSs, but all drugs alone could be fully amorphized. While the stability of each drug varied depending on the PLs used, the SE CAPS consistently demonstrated the highest stability by a significant margin. Initially, a 1:1 molar ratio was used for screening all systems, though the optimal molar ratio for drug stability remained uncertain. To address this, various molar ratios were investigated to determine the ratio yielding the highest amorphous drug stability. Our results indicate that all systems remained physically stable at a 1.5:1 ratio and with excess of PL. Furthermore, the CAPS formed by the SE significantly improves the dissolution behavior of CCX and RIT, whereas the PLs provide a slight precipitation inhibition for supersaturated CCX and RIT. **Conclusions**: These findings support the use of a 1:1 molar ratio in screening processes and suggest that CAPSs can be effectively prepared with relatively high drug loads compared to traditional drug–polymer systems. Furthermore, the study highlights the critical role of drug selection, the preparation method, and the PL type in developing stable and effective CAPSs.

## 1. Introduction

In recent studies, co-amorphous systems have emerged as a promising alternative to polymer-based amorphous solid dispersions [1]. Unlike polymer-based systems, co-amorphous materials demonstrate optimal drug-to-co-former ratios, often at a 1:1 molar ratio, reducing the need for high excipient concentrations [1,2]. Phospholipids (PL) exhibit favorable properties, making them suitable co-formers for poorly water-soluble drugs. They are Generally Recognized as Safe (GRAS), making them suitable for translation from bench to bedside. Additionally, PLs can interact with drugs through ionic, polar, and non-polar interactions, facilitating the conversion of crystalline drugs into an amorphous form [3]. Many studies have shown that drug–phospholipid systems are beneficial in enhancing the solubility, permeability, and oral bioavailability of the drug [4,5].

In a previous study [6], we explored the formation of co-amorphous drug–phospholipid systems (CAPSs) using the model drug indomethacin and three different phospholipids and preparation methods. Soybean phosphatidylcholine (SPC), hydrogenated phosphatidylcholine (HPC), and mono-acyl phosphatidylcholine (MAPC) were used as examples of unsaturated and saturated phospholipids, as well as lysophospholipids. The preparation methods employed were ball milling (as a standard method for mechanical energy input for amorphization), quench cooling (as a standard method for heat energy input for amorphization), and rotary solvent evaporation (as a standard method for solvent evaporation for amorphization). The results indicated the successful formation of co-amorphous systems using indomethacin (a drug easily converted to an amorphous form), providing valuable insights into the versatility of these phospholipids for CAPS preparation. Building on this previous case study, the current study aims to investigate CAPS formation by examining four additional drugs in combination with SPC, HPC, and MAPC. Shifting the focus from indomethacin to a broader range of pharmaceutical compounds (celecoxib, furosemide, nilotinib, and ritonavir), we aim to arrive at guidelines with respect to a generally recommendable choice of preparation method and PL type when aiming to develop CAPSs, as well as to increase our understanding of the preferential physicochemical properties of different drugs to develop CAPSs and their dissolution and supersaturation behavior [4,5,7]. This research is particularly interesting for drug formulation and delivery, where a time- and resource-sparing approach to developing stable co-amorphous systems that can potentially improve solubility, bioavailability, and therapeutic efficacy is desired.

We initially maintained an equimolar ratio of drug to PL (1:1), as most drug-PL complexes that have previously been investigated have used this molar ratio [5,7]. Additionally, we investigate a broader range of molar ratios spanning from 10:1 to 1:10 to assess the propensity of various drugs to form co-amorphous systems with SPC, HPC, and MAPC, again with a view to arriving at general recommendations with respect to their physical stability. The selected drugs exhibit a range of physicochemical properties, allowing for an evaluation of these factors influencing CAPS formation.

## 2. Materials and Methods

### 2.1. Materials

SPC (Mw = 782.0 g/mol) (Lipoid^®^ S 100, purity 98.9%), HPC (Mw = 790.0 g/mol) (PHOSPHOLIPON^®^ 90H, purity 95.8%), and MAPC (Mw = 508.0 g/mol) (Lipoid^®^ S LPC 80, purity 80%) were kindly donated by Lipoid GmbH (Ludwigshafen, Germany). Celecoxib (CCX) (Mw = 381.4 g/mol) was obtained from Dr Reddy’s (Hyderabad, India). Furosemide (FUR) (Mw = 330.7 g/mol) was purchased from Sigma Aldrich (St. Louis, MO, USA). IND (Mw = 357.8 g/mol) was obtained from Fagron A/S (Copenhagen, Denmark). Nilotinib (NIL) (Mw = 529.5 g/mol) was purchased from Huichem Co., Ltd. (Shanghai, China). Ritonavir (RIT) (Mw = 720.9 g/mol) was purchased from Nanjing Yanst Bio-Tech Co., Ltd. (Nanjing, China). Ethanol (Ph. Eur. grade), methanol (Ph. Eur. grade), acetonitrile (ACN) (Ph. Eur. grade), and dichloromethane (Ph. Eur. grade) were obtained from VWR (Herlev, Denmark). Trifluoroacetic acid (TFA) (HPLC grade) was obtained from Sigma Aldrich (Søborg, Denmark). Fasted state simulated intestinal fluid (FaSSIF v1) powder was purchased from Biorelevant Ltd. (London, UK).

### 2.2. Preparation of Physical Mixtures

Physical mixtures (PM) of the various drug–phospholipid systems were prepared by mixing and grinding the phospholipid and the drug component. In total, 500 mg of PMs were used, except for the NIL-PL systems where 200 mg were used. Different molar ratios of drug/PL (10:1, 5:1, 3:1, 2:1, 1.5:1, 1:1, 1:1.5, 1:2, 1:3, 1:5, and 1:10) were weighed and mixed using a mortar and pestle twice for 60 s. The impurity levels of the phospholipids were considered (as detailed in Section 2.1), and their purity was factored into the calculation of equal ratios. The mixtures were stored at −20 °C. All preparations were prepared in independent triplicates (*n* = 3).

### 2.3. Ball Milling

Ball milling (BM) was employed to prepare various drug–phospholipid mixtures. The ball mill (Mixer Mill MM400, Retsch GmBH & Co., Haan, Germany) was operated in a cold room set at 6 °C. PMs were placed in 5 mL jars, each containing 200 mg of the mixture and a single 5 mm diameter stainless steel ball. The mixtures were milled for 120 min at a frequency of 30 Hz (except for FUR-PLs, which were only milled for 60 min due to visible degradation upon longer milling times). BM was stopped every 10 min and not restarted until the milling jars reached a temperature below 10 °C, measured with an infrared thermometer. All samples were prepared in independent triplicates (*n* = 3).

### 2.4. Quench-Cooling

PMs were evenly distributed on aluminum foils and heated either on a hotplate or in an oven, both set to 10 °C above the drugs’ melting points, CCX (163 °C), FUR (219 °C), NIL (232 °C), and RIT (126 °C). Upon visual confirmation of complete melting, the melts were stirred with a spatula to ensure a uniform mixture and prevent phase separation. However, FUR and NIL required stirring at the onset of melting due to the drugs’ melting points being close to their onset degradation temperatures. Subsequently, the mixtures were cooled to room temperature (21 °C) and gently ground using a mortar and pestle. All preparations were prepared in independent triplicates (*n* = 3).

### 2.5. Rotary Solvent Evaporation

PMs were dissolved in suitable solvents or solvent combinations at room temperature, with a batch volume of 30 mL (Table 1). These solutions were prepared under magnetic stirring and sonicated for 30 min to achieve a homogenous solution. The solutions were then transferred to round-bottom flasks, and the solvent was evaporated using a rotary evaporator at 40 °C, adhering to the vapor pressures detailed in Table 1 to avoid foam formation. After most of the solvent was evaporated, the pressure was reduced to 10 mbar for 30 min to complete the evaporation. Finally, the mixtures were left overnight in a vacuum chamber at 10 mbar to remove the remaining solvent residues. All samples were prepared in independent triplicates (*n* = 3).

### 2.6. X-ray Powder Diffraction

XRPD analysis was conducted on initial materials and various mixtures using an X’Pert PRO diffractometer (PANalytical, Almelo, The Netherlands) with Cu Kα radiation (λ = 1.5406 Å) at 45 kV and 40 mA. Samples were placed on aluminum sample holders and scanned over an angular range of 5–35° 2θ at a scanning speed of 0.058° 2θ/min and a step size of 0.026° 2θ. Diffractograms were analyzed using X’Pert HighScore Plus (v2.2.4) software (PANalytical, Almelo, The Netherlands). Measurements were performed in triplicate (*n* = 3).

### 2.7. Polarized Light Microscopy

Polarized light microscopy (PLM) was conducted on a Leica DM LM microscope (Leica Microsystems GmbH, Wetzlar, Germany) equipped with an Evolution MP Camera (Media Cybernetics, Rockville, MD, USA) controlled by the Image-Pro Insight software version 8.0 (Media Cybernetics, Rockville, MD, USA). The microscope was operated in both transmitted-light and transmitted cross-polarized-light modes, where the light was produced by a halogen lamp (12 V, 100 W). The samples were spread and squeezed evenly between glass coverslips to observe possible birefringent textures at a 10× objective magnification. Measurements were performed in triplicates (*n* = 3).

### 2.8. Differential Scanning Calorimetry

Thermal analysis was carried out using a Discovery differential scanning calorimeter (TA Instruments Inc., New Castle, DE, USA). Samples weighing 4–8 mg were analyzed in Tzero aluminum pans sealed with perforated lids. The furnace was purged with nitrogen gas at a flow rate of 50 mL/min. The DSC instrument was calibrated for temperature and enthalpy using indium as a standard. The melting point (T_m_, onset of the endothermic event) and glass transition temperatures (T_g_, midpoint of the step change heat flow) were determined using the TRIOS software v5.1.1 (TA Instruments Inc., New Castle, DE, USA). The samples were heated from 20 °C below their T_g_ to 5–10 °C above the drug’s T_m_ at a heating rate of 10 °C/min. Measurements were performed in triplicates (*n* = 3).

### 2.9. Physical Stability Studies

Physical stability studies were performed on the pure amorphous drugs, the drug–phospholipid mixtures, which formed CAPSs, and on drug-PL systems in which only the drug became amorphous. The samples were stored in desiccators close to 0% relative humidity (RH) using phosphorus pentoxide (P_2_O_5_). In addition, a higher RH of 75%, achieved by using a saturated sodium chloride solution, was used at room temperature to investigate the physical stability of CCX-phospholipid and RIT-phospholipid samples at different molar ratios. The physical stability of the CAPSs was investigated by XRPD, PLM, and DSC. The first occurrence of Bragg peaks in the XRPD diffractograms, birefringence in the PLM originating from the drug in the CAPS, and endothermic melting events in the DSC thermograms were deemed the time point of recrystallization. The mixtures were analyzed weekly for the first month, then every 15 days (±5 days) until drug recrystallization occurred. For RIT, samples were investigated for 180 days.

### 2.10. Determination of Equilibrium Solubility

The equilibrium solubility (S_eq_) of various crystalline drugs in a fasted-state simulated intestinal fluid (FASSIF v1) was determined by adding an excess of the drug to 1 mL of FASSIF v1. Suspensions were placed on an RCT basic rotator system (IKA, Staufen, Germany) at 37 °C. After 24 h, a clear supernatant was obtained by centrifugation at 19,000× *g* for 15 min at 25 °C, followed by appropriate dilution with acetonitrile containing 0.05% TFA. Drug content was quantified using HPLC as detailed in Section 2.12. All preparations and measurements were performed independently in triplicate (*n* = 3).

### 2.11. In Vitro Dissolution

In vitro dissolution studies were conducted under non-sink conditions using a miniaturized USP2 apparatus with 100 mL FaSSIF v1 (pH 6.5, 37 °C) as the dissolution medium. To address the poor wetting and dispersibility of the co-amorphous drug–phospholipid systems, an initial pre-dispersion step was carried out. A total of 10 mg of the drug was pre-dispersed in 10 mL of FaSSIF v1 and vortexed for 30 s. The studies were initiated by transferring the pre-dispersed mixtures into 200 mL glass vials containing 90 mL of FaSSIF v1. At designated time points (1, 5, 10, 15, 20, 30, 45, 60, 120, and 240 min), 2 mL aliquots were filtered through syringe filters (0.45 µm membrane). The initial 1 mL of filtrate was discarded, and the remaining 1 mL was collected and centrifuged at 13,000× *g* for 20 min at room temperature. The drug content in the resulting samples was then quantified using HPLC. All preparations and analyses were performed independently in triplicate (*n* = 3).

### 2.12. High-Performance Liquid Chromatography

Quantification of the various drugs was conducted by HPLC coupled to a UV detector using an Agilent 1260 Infinity chromatographic system (Agilent Technologies, Santa Clara, CA, USA) equipped with an Agilent 1290 Diode Array Detector. A Phenomenex^®^ C18 (4.60 mm × 150 mm, 5 µm) column (Torrance, CA, USA) was used. An analysis of the different systems was performed, as shown in Table 2.

### 2.13. Statistical Analysis

Statistical analysis between more than two groups was determined by one-way ANOVA (*p* = 0.05) followed by Tukey’s multiple comparisons test using GraphPad Prism (software version 10.1.2), San Diego, CA, USA.

## 3. Results and Discussion

This study builds upon our prior investigation of forming CAPSs with indomethacin and PLs, SPC, HPC, and MAPC using BM, QC, and SE as preparation methods [6]. Expanding upon this, we now explore CAPS formation with a range of poorly water-soluble model drugs, including celecoxib (CCX), furosemide (FUR), nilotinib (NIL), and ritonavir (RIT) (Table 3). An equimolar ratio of drug to PL (1:1) was maintained throughout this part of the study to ensure a standardized assessment across the various drug-PL combinations. By employing the same preparation methods, our investigation examines the impact of both the drug and the preparation techniques on the formation of CAPSs.

### 3.1. Solid-State Characterization of Model Drugs and Co-Amorphous Drug-PL Systems

#### 3.1.1. Model Drugs

Initially, the model drugs were investigated as received. The analysis was conducted following the methodology outlined in our previous case study [6]. Three key observations indicate the successful amorphization of the drug: the absence of Bragg peaks in the XRPD diffractograms, birefringence under polarized light, and the presence of a T_g_, and absence of a melting endotherm in the DSC thermograms [14,15,16]. Table 4 summarizes the findings for the pure drugs, CCX (Figure S1), FUR (Figure S2), NIL (Figure S3), and RIT (Figure S4) produced by the three preparation methods, BM, QC, and SE.

The results demonstrate that the different preparation methods successfully amorphized pure CCX, FUR, and RIT. NIL turned amorphous by BM and QC, whereas this was not feasible by SE with the solvents and the experimental setup outlined in Section 2.5. These findings show that the chosen drugs, except SE NIL, can become amorphous on their own by the chosen preparation methods. This aligns with their GFA classifications: CCX, FUR, NIL (GFA Class 2), and RIT (GFA Class 3).

#### 3.1.2. Phospholipids

Confirming the results of our previous case study, the solid-state characterization of the PLs (SPC, HPC, and MAPC) indicated that converting and maintaining bulk phospholipids in an amorphous form following BM, QC, and SE is not feasible with the chosen method parameters [6].

#### 3.1.3. Solid State Characterization of Equimolar Drug–Phospholipid Systems

All equimolar drug-SPC systems (Table 5) prepared by BM displayed conversion of the initially crystalline drug into amorphous forms, while the SPC remained crystalline. Preparation by QC successfully formed CAPSs for CCX-SPC (Figure S5) and RIT-SPC (Figure S6). In contrast, the FUR-SPC (Figure S7) and NIL-SPC (Figure S8) systems did not become co-amorphous due to the drugs’ degradation temperature being too close to their melting point. The systems prepared by SE resulted in CAPSs in the case of CCX, FUR, and RIT-SPC systems, whereas the NIL-SPC system did not become amorphous.

CCX-HPC (Figure S9) and RIT-HPC (Figure S10) systems prepared by BM resulted in an amorphous drug (Table 5), but HPC remained crystalline. In contrast, the FUR-HPC (Figure S11) and NIL-HPC (Figure S12) systems remained crystalline. On the other hand, preparation by QC resulted in amorphous CCX but crystalline HPC. FUR-HPC and NIL-HPC did not form CAPSs because the drugs’ degradation temperature was too close to their T_m_. QC successfully formed co-amorphous RIT-HPC. For SE, CCX-HPC, FUR-HPC, and RIT-HPC systems resulted in CAPSs, whereas NIL-HPC remained crystalline.

The drug-MAPC systems followed a similar behavior to the drug-HPC systems. CCX-MAPC (Figure S13) and RIT-MAPC (Figure S14) systems prepared by BM resulted in an amorphous drug (Table 5), but MAPC remained crystalline. In contrast, FUR-MAPC (Figure S15) and NIL-MAPC (Figure S16) systems remained crystalline. QC successfully formed CCX-MAPC and RIT-MAPC CAPSs, in contrast, FUR-MAPC and NIL-MAPC did not form CAPSs because of the drugs’ degradation temperature being too close to their T_m_. For SE, CCX-MAPC, FUR-MAPC, and RIT-MAPC systems resulted in CAPSs, whereas NIL-MAPC remained crystalline.

### 3.2. Influence of Drug Properties on the Formation of Co-Amorphous Drug–Phospholipid System

CAPSs were formed with the drugs that demonstrated an ability to become amorphous as pure drugs by SE, namely CCX, FUR, and RIT. These drugs exhibit LogP values within 1.75–5.22, Mw between 330.7 and 720.9 g/mol, T_m_ ranging from 126 to 219 °C, and T_g_ within 45–78 °C (Table 3). In contrast, NIL’s drug properties did not fall within the ranges mentioned above, and it was not possible to either be converted into an amorphous form by SE or to form equimolar CAPSs. Gautschi et al. proposed that drugs with LogP values exceeding five and enthalpies of fusion greater than 150 J/g generally could not produce amorphous drug-MAPC systems at a 1:1 molar ratio [5]. Building on these findings, we propose a refined rule of thumb for screening model drugs to form equimolar CAPSs with SPC, HPC, and MAPC: the likelihood of CAPS formation is high if the drug properties fall within the following values: enthalpy of fusion < 150 J/g, and LogP < 5.2. Our results confirm thresholds suggested by Gautschi et al. indicating that specific drug properties are crucial for successfully forming CAPSs [5].

### 3.3. Influence of Preparation Methods on Co-Amorphous Drug–Phospholipid System Formation

The preparation method for CAPSs significantly influences their formation. BM of drug-PL systems resulted in the drugs becoming amorphous but the PLs remained crystalline. For QC drug-PL systems, all RIT-PL combinations formed co-amorphous systems, whereas CCX-HPC systems showed Bragg peaks and birefringence, indicating incomplete amorphization. In contrast, forming amorphous systems of FUR-PLs and NIL-PLs was not possible due to their T_m_’s proximity to degradation temperatures. SE was the most successful preparative method, achieving co-amorphization of CCX-PLs, FUR-PLs, and RIT-PLs. However, NIL remained crystalline, as evidenced by the presence of diffraction peaks.

These findings underscore that the choice of preparation method is crucial for the successful formation of CAPSs. SE emerged as the most promising method, followed by QC, with BM being the least favorable due to its inability to amorphize PLs under the studied conditions.

### 3.4. Influence of Phospholipid Type on the Formation of Co-Amorphous Drug–Phospholipid Systems

Out of the 12 systems investigated for each of the PLs, SPC systems (Table 5) led to 5 CAPSs, 4 systems with amorphous drug only, and 3 systems that remained crystalline. HPC systems (Table 5) led to four CAPSs, three systems with amorphous drug only, and five systems that remained crystalline. MAPC systems (Table 5) led to five CAPSs, two systems with amorphous drug only, and five systems that remained crystalline. Therefore, when developing CAPSs, all types of PLs should be tested but it is perhaps advisable to prioritize the use of SPC and MAPC over HPC.

### 3.5. Influence of Co-Amorphization on the Physical Stability of Amorphous Drug Forms

**Figure 1:**
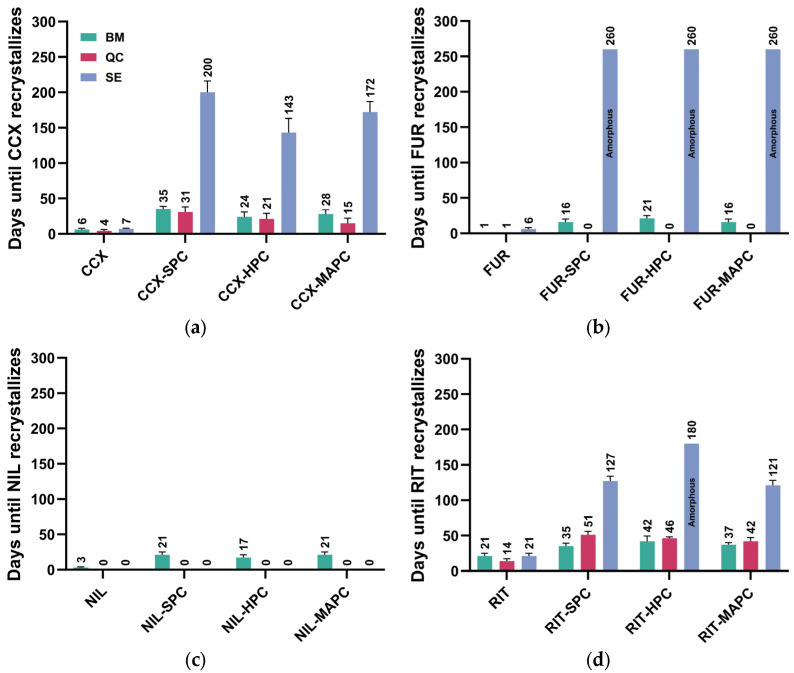
Physical stability of equimolar drug-PL systems prepared by ◼BM, ◼QC, and ◼SE after storage in 0% RH at RT. (**a**) CCX-PLs, (**b**) FUR-PLs, (**c**) NIL-PLs, and (**d**) RIT-PLs. Physical stability refers to the time until the drug crystallizes. Systems that did not become amorphous are labeled as “0 days”. Data are represented as mean ± SD (*n* = 3).

All systems were stored at 0% RH at RT and evaluated by XPRD, PLM, and DSC. As mentioned above, the systems were deemed recrystallized when the model drug had recrystallized. The physical stability data of equimolar CAPSs shows that the SE drug-PL systems with CCX, FUR, and RIT have higher physical stability than the pure amorphous drugs and the systems prepared by other preparation methods (Figure 1).

In contrast, none of the NIL-PL systems reached any appreciable physical stability. Whilst the stabilities varied for each drug depending on the phospholipid used, the SE CAPS by far showed the highest stability (Figure 1).

These findings prompted us to choose the solvent-based preparation method for the following experiments.

### 3.6. Screening for the Optimal Drug-PL Ratio

Initially, we used a 1:1 molar ratio for screening all systems. However, it remains unclear whether this is the best drug-PL ratio. Therefore, in this part of the study, we investigated different molar ratios to identify the drug-PL ratio with the highest amorphous drug stability.

**Figure 2:**
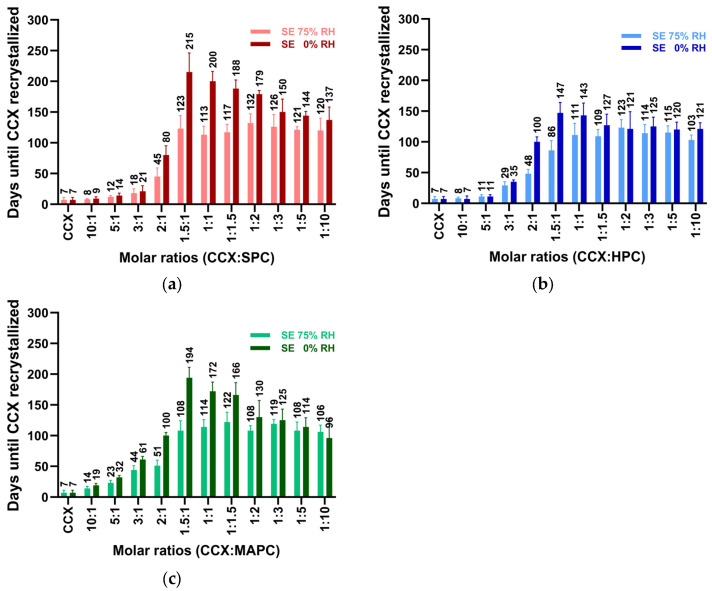
Physical stability of various molar ratios of CCX:PL systems at 75% RH and 0% RH at RT, prepared by SE. (**a**) CCX-SPC, (**b**) CCX-HPC, and (**c**) CCX-MAPC. Data are represented as mean ± SD (*n* = 3). Physical stability refers to the time until the drug crystallizes.

This was investigated using the model drugs CCX and RIT at various molar ratios with SPC, HPC, and MAPC. The physical stability of these formulations was evaluated at room temperature under two distinct humidity conditions: 75% relative humidity (RH) and 0% RH. Stability, assessed using XRPD, PLM, and DSC, was determined as the time until drug recrystallization occurred. As shown in Figure 2, the physical stability of the CCX-PL systems varied significantly depending on the humidity and specific PL utilized. As expected, the formulations consistently displayed superior stability in all PL types, when stored under 0% RH conditions compared to 75% RH [3,17]. The data show that the most stable co-amorphous CCX-PL systems are formed at a 1.5:1 molar ratio or with an excess PL under both humidity conditions and with different PLs.

**Figure 3:**
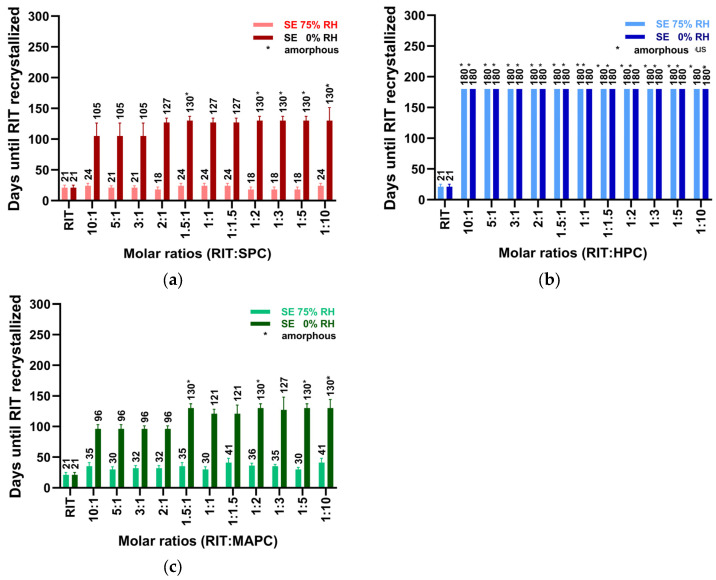
Physical stability of various molar ratios of RIT:PL systems at 75% RH and 0% RH at RT, prepared by SE. (**a**) RIT-SPC, (**b**) RIT-HPC, and (**c**) RIT-MAPC Data are represented as mean ± SD (*n* = 3). Physical stability refers to the time until the drug crystallizes.

The stability of the RIT systems is shown in Figure 3a–c. RIT-SPC and RIT-MAPC systems also displayed higher physical stability when stored at 0% relative humidity compared to 75%. Notably, at 75% RH, these formulations exhibited only 1/3 of the physical stability observed at 0% RH. In contrast, the RIT-HPC system (Figure 3b) remained co-amorphous throughout the stability testing, spanning 180 days, regardless of humidity. The data show a slightly higher physical stability of RIT-SPC and RIT-MAPC systems with molar ratios above 1.5:1, whereas, the RIT-HPC systems all remained amorphous throughout the 180-day physical stability study.

In general, all systems showed the highest stability at around 1.5:1 (with the 1:1 and 1:1.5 ratios not being significantly different). These findings rationalize the use of a 1:1 molar ratio in screening processes and indicate that these forms of co-amorphous systems can be prepared at higher drug loads compared to polymer-based amorphous solid dispersions [1].

### 3.7. Influence of Phospholipids on the Dissolution Behavior of CCX-PL and RIT-PL

Having established SE as the preferred preparation method and the drug-PL ratios of 1:1 as the preferred ratio for amorphous stability, the next step involved evaluating the dissolution behavior of CCX and RIT CAPSs using non-sink in vitro dissolution testing.

**Figure 4:**
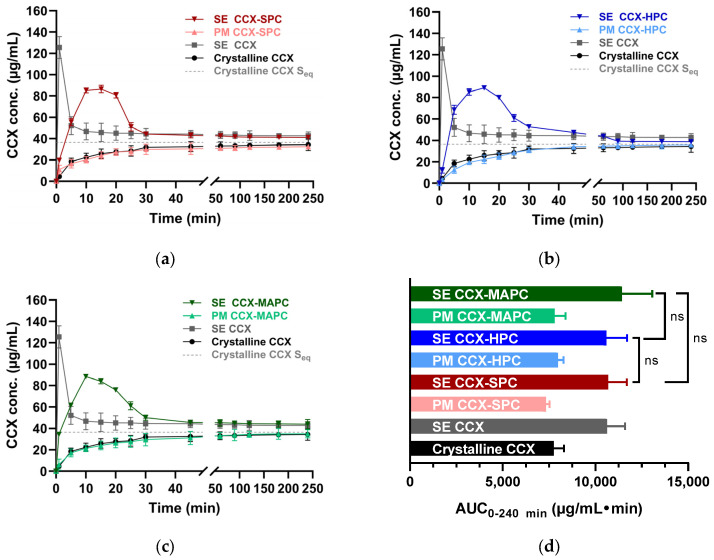
In vitro non-sink dissolution profiles of crystalline CCX, amorphous SE CCX, and equimolar CCX-PL systems in FaSSIF v1 (pH 6.5) at non-sink conditions for 0–240 min. (**a**) CCX-SPC systems (**b**) CCX-HPC systems (**c**) CCX-MAPC systems. (**d**) Corresponding AUC_0–240 min_ of (**a**–**c**). The AUC_0–240 min_ for SE CCX-SPC, SE CCX-HPC, and SE CCX-MAPC showed no significant difference, as indicated by ‘ns’. Data are represented as mean ± SD (*n* = 3).

The dissolution profiles of amorphous CCX exhibited a high initial dissolution rate, rapidly leading to supersaturation. This was quickly followed by precipitation within 1 min, bringing the CCX concentration down to approximately its equilibrium solubility (37 µg/mL) at 10 min. The PMs of crystalline CCX-PLs show that the simple addition of the PLs to the crystalline drug did not influence the dissolution profiles (Figure 4).

The dissolution profiles of the CCX CAPSs exhibited a more gradual increase in the dissolved CCX concentration, reaching approx. 90 µg/mL within 10–20 min. Gradual precipitation was observed hereafter until the dissolved drug concentrations stabilized at the equilibrium solubility of CCX (37 µg/mL) at approx. 30 min. The area under the dissolution curve (AUC) for amorphous CCX and CCX-PL CAPSs were not significantly different (Figure 4d), indicating that the PL did not increase the overall amount of dissolved drug, but that the PL exhibited a slight precipitation-inhibiting effect. In contrast, the AUC for crystalline CCX and physical mixtures of CCX-PLs differed from the SE samples, confirming that SE (and thus CAPS formation) improved drug dissolution behavior.

Thus, it can be concluded that formed CAPSs of CCX-PL enhance the dissolution, and PLs provide a slight precipitation inhibition for supersaturated CCX.

Previous studies on drug-PL complexes have also shown an increase in solubility and drug dissolution compared to the crystalline drug [15,18,19,20,21].

**Figure 5:**
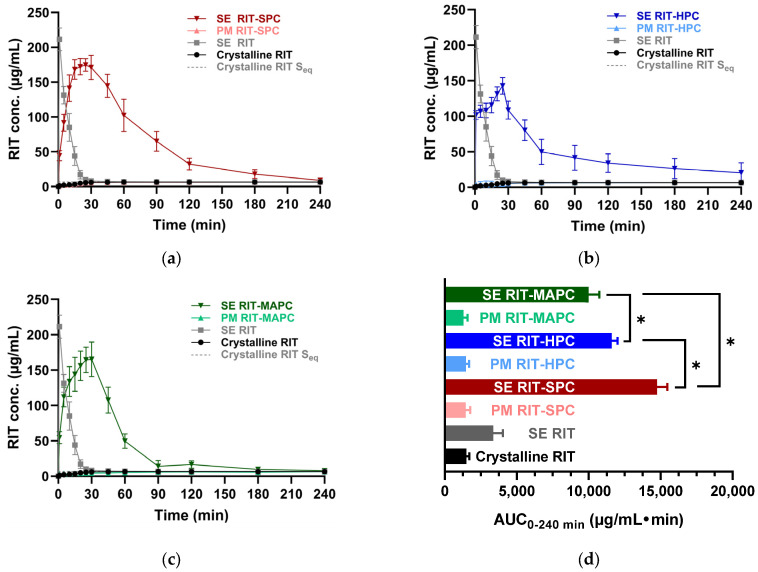
In vitro non-sink dissolution profiles of crystalline RIT, amorphous SE RIT, and equimolar RIT-PL systems in FaSSIF v1 (pH 6.5) at non-sink conditions for 0–240 min. (**a**) RIT-SPC systems, (**b**) RIT-HPC systems, (**c**) RIT-MAPC systems. (**d**) Corresponding AUC_0–240 min_ of (**a**–**c**). The AUC_0–240 min_ for SE RIT-SPC, SE RIT-HPC, and SE RIT-MAPC showed a significant difference, indicated by the asterisk (*). Data are represented as mean ± SD (*n* = 3).

As for amorphous CCX, the dissolution profiles of amorphous RIT showed a high initial dissolution rate leading to supersaturation, followed by precipitation (after 1 min) (Figure 5), resulting in RIT reaching a concentration close to its equilibrium solubility (6.7 µg/mL) right before 30 min. The crystalline PMs again show that the physical addition of the PLs had no significant influence on the dissolution profile for crystalline RIT. On the other hand, the dissolution profiles of the RIT-PL CAPS showed a more gradual increase in the dissolved RIT concentration, reaching supersaturation at approx. 20 min, followed by gradual precipitation until reaching concentrations equal to the equilibrium solubility of the drug (Figure 5). The AUC_0–240 min_ shows that the RIT-SPC CAPS showed significantly higher AUC than the RIT-HPC and RIT-MAPC CAPSs.

Thus, it can be concluded that the CAPS formed by SE greatly enhance the dissolution behavior of the drugs, but that PLs in the CAPS only provide a slight precipitation inhibition for supersaturated RIT.

## 4. Conclusions

This study investigated various drugs, preparation methods, and PL types for CAPS preparation, evaluating their effectiveness in terms of physical stability and drug dissolution. Overall, the findings indicate that the previously determined physicochemical preferred properties of the drugs for CAPS formation could be confirmed. With respect to stability, SE emerged as the preferred preparation method. With respect to PL type, no major differences were seen for the formation of CAPSs, but a drug-dependent effect of the PL type with respect to physical stability was observed, with CCX-PLs, FUR-PLs, and RIT-PLs resulting in significantly higher physical stability than their crystalline counterpart. CAPSs led to a higher drug dissolution compared to crystalline drugs and PMs and exhibited some precipitation inhibition compared to the pure amorphous drug. However, this could be further improved by the addition of precipitation inhibitors.

## Figures and Tables

**Table 1:** Composition of solvent(s) used for rotary solvent evaporation (SE) of the different drug-PL systems.

Systems	Ethanol (v%)	Methanol (v%)	Dichloromethane (v%)	Vapor Pressure (mbar)
CCX-PL	100	-	-	170
FUR-PL	-	100	-	130
NIL-PL	-	50	50	250–130
RIT-PL	70	30	-	170

**Table 2:** Parameters and mobile phases employed for the quantification of CCX-, FUR-, NIL-, and RIT-systems.

Systems	Injection Volume (µL)	Wavelength (nm)	Column, T (°C)	Flow Rate (mL/min)	Mobile Phase A (*v*/*v*)	Mobile Phase B (*v*/*v*)
CCX-PL	10	251	40	1.0	Methanol 75%	Water 25%
FUR-PL	20	275	25	1.0	Methanol 65%	Water + 0.1%TFA 35%
NIL-PL	10	267	40	1.2	Water + 0.1% TFA 90% 0–3 min 90→10% 3–6 min 10% 6–8 min 10→90% 8–12 min 90% 12–18 min	ACN + 0.1%TFA 10% 0–3 min 10→90% 3–6 min 90% 6–8 min 90→10% 8–12 min 10% 12–18 min
RIT-PL	10	213	25	1.0	ACN 60% (*v*/*v*)	Water 40% (*v*/*v*)

**Table 3:** Physical properties [8], biopharmaceutics classification system (BCS), and glass forming ability (GFA) of the model drugs [9,10,11].

Drug	M_W_ (g/mol)	Enthalpy of Fusion ^a^ (J/g)	T_m_^c^ (°C)	T_g_^d^ (°C)	LogP ^b^	BCS Class	GFA Class ^e^	Poly-Morphic Form
Celecoxib	381.4	87.3	163	51	4.0	II	2	III
Furosemide	330.7	63.7	219	78	1.8	IV	2	I
Nilotinib	529.5	136.0	232	104	4.5	IV	2	I
Ritonavir	720.9	73.1	126	50	5.2	II	3	II

M_W_ values are taken from the Cambridge Crystallographic Data Center. ^a^ values determined by DSC; ^b^ values from Chemaxon; ^c^ values are taken as the onset of the melting endotherm; ^d^ values are taken as the average midpoint of T_g_; ^e^ Celecoxib [12], furosemide [13], nilotinib, and ritonavir were determined following the same procedure described in Blaabjerg et al. 2016 [12].

**Table 4:** Characterization of model drugs prepared by BM, QC, and SE. Diffractograms, micrographs, and thermograms were used to identify if the model drugs were amorphous (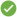) or crystalline (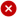).

CCX			FUR			NIL			RIT		
BM	QC	SE	BM	QC	SE	BM	QC	SE	BM	QC	SE
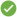	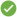	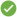	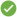	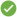	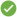	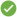	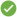	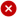	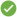	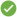	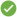

**Table 5:** Characterization of equimolar drug-SPC systems prepared by BM, QC, and SE. PLM, XRPD, and DSC were used to identify whether samples were co-amorphous (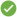), showed only crystallinity of SPC (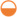), or both drug and SPC crystalline (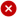).

**CCX-SPC**			**FUR-SPC**			**NIL-SPC**			**RIT-SPC**		
BM	QC	SE	BM	QC	SE	BM	QC	SE	BM	QC	SE
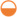	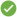	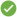	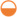	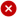	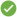	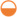	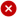	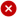	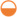	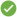	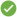
**CCX-HPC**			**FUR-HPC**			**NIL-HPC**			**RIT-HPC**		
BM	QC	SE	BM	QC	SE	BM	QC	SE	BM	QC	SE
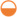	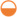	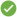	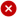	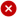	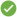	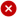	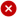	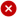	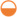	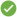	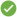
**CCX-MAPC**			**FUR-MAPC**			**NIL-MAPC**			**RIT-MAPC**		
BM	QC	SE	BM	QC	SE	BM	QC	SE	BM	QC	SE
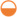	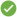	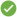	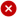	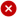	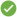	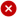	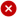	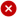	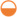	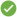	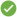

## Data Availability

The original contributions presented in the study are included in the article/Supplementary Materials, further inquiries can be directed to the corresponding author.

## Supplementary Materials

The following supporting information can be downloaded at: https://www.mdpi.com/article/10.3390/pharmaceutics16121602/s1, Figure S1: Solid state characterization, XRPD, PLM, and DSC of CCX; Figure S2: Solid state characterization, XRPD, PLM, and DSC of FUR; Figure S3: Solid state characterization, XRPD, PLM, and DSC of NIL; Figure S4: Solid state characterization, XRPD, PLM, and DSC of RIT; Figure S5: Solid state characterization, XRPD, PLM, and DSC of CCX-SPC; Figure S6: Solid state characterization, XRPD, PLM, and DSC of RIT-SPC; Figure S7: Solid state characterization, XRPD, PLM, and DSC of FUR-SPC; Figure S8: Solid state characterization, XRPD, PLM, and DSC of NIL-SPC; Figure S9: Solid state characterization, XRPD, PLM, and DSC of CCX-HPC; Figure S10: Solid state characterization, XRPD, PLM, and DSC of RIT-HPC; Figure S11: Solid state characterization, XRPD, PLM, and DSC of FUR-HPC; Figure S12: Solid state characterization, XRPD, PLM, and DSC of NIL-HPC; Figure S13: Solid state characterization, XRPD, PLM, and DSC of CCX-MAPC; Figure S14: Solid state characterization, XRPD, PLM, and DSC of RIT-MAPC; Figure S15: Solid state characterization, XRPD, PLM, and DSC of FUR-MAPC; Figure S16: Solid state characterization, XRPD, PLM, and DSC of NIL-MAPC.

## References

[1] Liu J., Grohganz H., Löbmann K., Rades T., Hempel N.J. (2021). Co-Amorphous Drug Formulations in Numbers: Recent Advances in Co-Amorphous Drug Formulations with Focus on Co-Formability, Molar Ratio, Preparation Methods, Physical Stability, In Vitro and In Vivo Performance, and New Formulation Strategies. Pharmaceutics.

[2] Rades T., Gordon K.C., Graeser K. (2013). Molecular Structure, Properties, and States of Matter. Remington: Essentials of Pharmaceutics.

[3] van Hoogevest P. (2017). Review—An update on the use of oral phospholipid excipients. Eur. J. Pharm. Sci..

[4] Fong S.Y.K., Ibisogly A., Bauer-Brandl A. (2015). Solubility enhancement of BCS Class II drug by solid phospholipid dispersions: Spray drying versus freeze-drying. Int. J. Pharm..

[5] Gautschi N., Van Hoogevest P., Kuentz M. (2017). Molecular insights into the formation of drug-monoacyl phosphatidylcholine solid dispersions for oral delivery. Eur. J. Pharm. Sci..

[6] Khorami K., Müllertz A., Rades T. (2024). Influence of preparation method and choice of phospholipid on co-amorphization, physical stability, and dissolution behavior of equimolar indomethacin-phospholipid systems—A case study. J. Drug Deliv. Sci. Technol..

[7] Gautschi N., Van Hoogevest P., Kuentz M. (2015). Amorphous drug dispersions with mono and diacyl lecithin: On molecular categorization of their feasibility and UV dissolution imaging. Int. J. Pharm..

[8] Baird J.A., Van Eerdenbrugh B., Taylor L.S. (2010). A classification system to assess the crystallization tendency of organic molecules from undercooled melts. J Pharm Sci.

[9] Markovic M., Zur M., Ragatsky I., Cvijić S., Dahan A. (2020). BCS Class IV Oral Drugs and Absorption Windows: Regional-Dependent Intestinal Permeability of Furosemide. Pharmaceutics.

[10] Xia B., Heimbach T., He H., Lin T.-H. (2012). Nilotinib preclinical pharmacokinetics and practical application toward clinical projections of oral absorption and systemic availability. Biopharm. Drug Dispos..

[11] Jitta S.R., Salwa, Bhaskaran N.A., Marques S.M., Kumar L. (2022). Recent advances in nanoformulation development of Ritonavir, a key protease inhibitor used in the treatment of HIV-AIDS. Expert Opin. Drug Deliv..

[12] Blaabjerg L.I., Lindenberg E., Löbmann K., Grohganz H., Rades T. (2016). Glass Forming Ability of Amorphous Drugs Investigated by Continuous Cooling and Isothermal Transformation. Mol. Pharm..

[13] Blaabjerg L.I., Lindenberg E., Rades T., Grohganz H., Löbmann K. (2017). Influence of preparation pathway on the glass forming ability. Int. J. Pharm..

[14] Hancock B.C., Zografi G. (1997). Characteristics and significance of the amorphous state in pharmaceutical systems. J. Pharm. Sci..

[15] Guo B., Liu H., Li Y., Zhao J., Yang D., Wang X., Zhang T. (2014). Application of phospholipid complex technique to improve the dissolution and pharmacokinetic of probucol by solvent-evaporation and co-grinding methods. Int. J. Pharm..

[16] Ma X., Williams R.O. (2019). Characterization of amorphous solid dispersions: An update. J. Drug Deliv. Sci. Technol..

[17] Hancock B.C., Zografi G. (1994). The Relationship Between the Glass Transition Temperature and the Water Content of Amorphous Pharmaceutical Solids. Pharm. Res..

[18] Singh D., Rawat M.S., Semalty A., Semalty M. (2012). Rutin-phospholipid complex: An innovative technique in novel drug delivery system-NDDS. Curr. Drug Deliv..

[19] Amirinejad M., Davoodi J., Abbaspour M.R., Akhgari A., Hadizadeh F., Badiee A. (2020). Preparation, characterization and improved release profile of ibuprofen-phospholipid association. J. Drug Deliv. Sci. Technol..

[20] Zhang K., Gu L., Chen J., Zhang Y., Jiang Y., Zhao L., Bi K., Chen X. (2015). Preparation and evaluation of kaempferol–phospholipid complex for pharmacokinetics and bioavailability in SD rats. J. Pharm. Biomed. Anal..

[21] Biswas S., Mukherjee P.K., Kar A., Bannerjee S., Charoensub R., Duangyod T. (2021). Optimized piperine–phospholipid complex with enhanced bioavailability and hepatoprotective activity. Pharm. Dev. Technol..

