# Supplementary material for: Drug–Phospholipid Co-Amorphous Formulations: The Role of Preparation Methods and Phospholipid Selection
Source: Pharmaceutics. 2024 Dec 16;16(12):1602. doi: 10.3390/pharmaceutics16121602 (PMC11676457; doi:10.3390/pharmaceutics16121602)
Supplement: Supplementary file 1 [file pharmaceutics-16-01602-s001.zip › pharmaceutics-3311137-supplementary.pdf]

SUPPLEMENTARY FIGURES

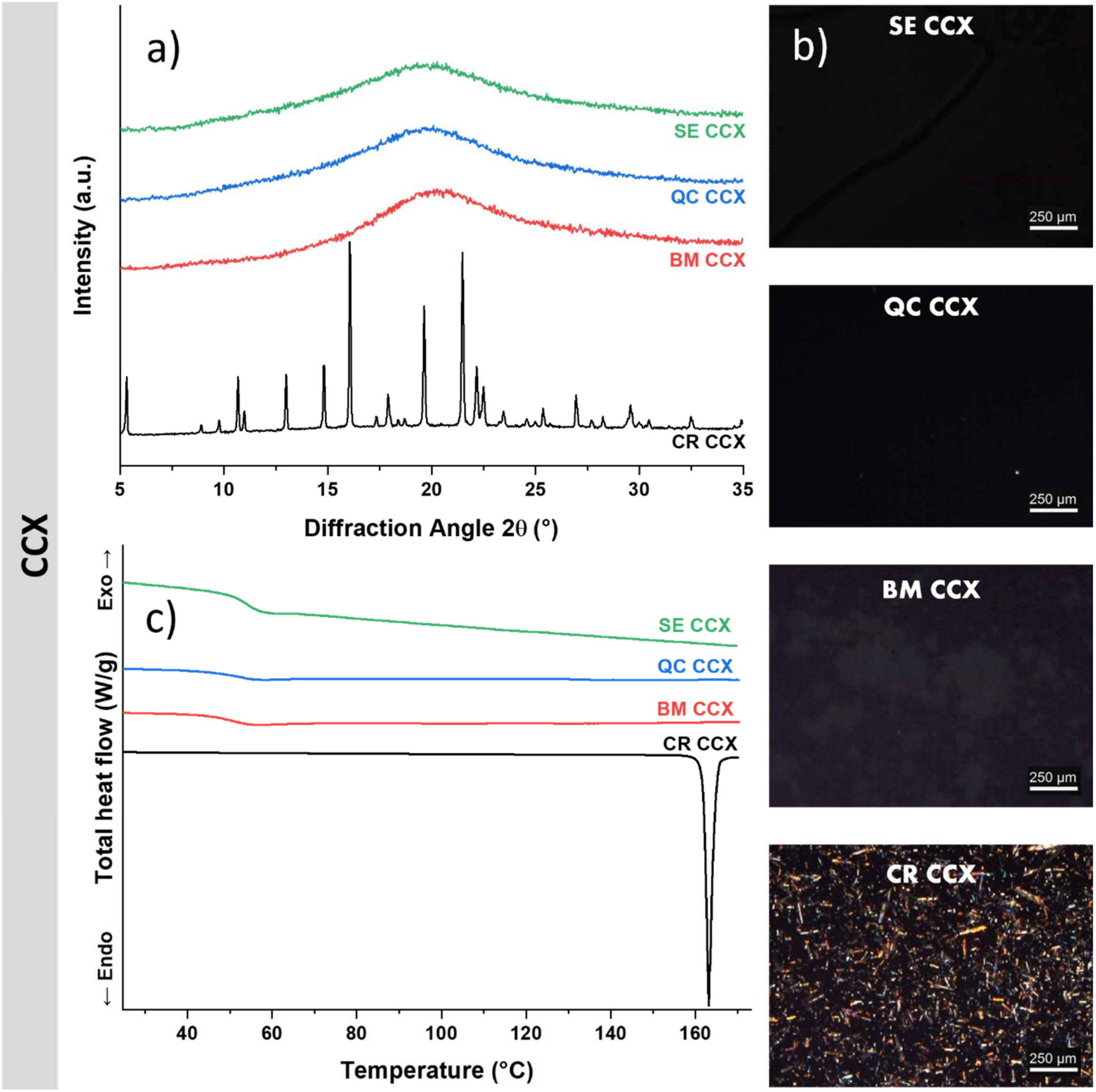

Figure S1. X-ray powder diffraction (XRPD) diffractograms (a), Polarized light microscopy (PLM) micrographs (b), and differential scanning calorimetry (DSC) thermograms (c) of celecoxib (CCX). Starting material crystalline (CR) CCX, and material after preparation by ball milling (BM), quench cooling (QC), and solvent evaporation (SE).

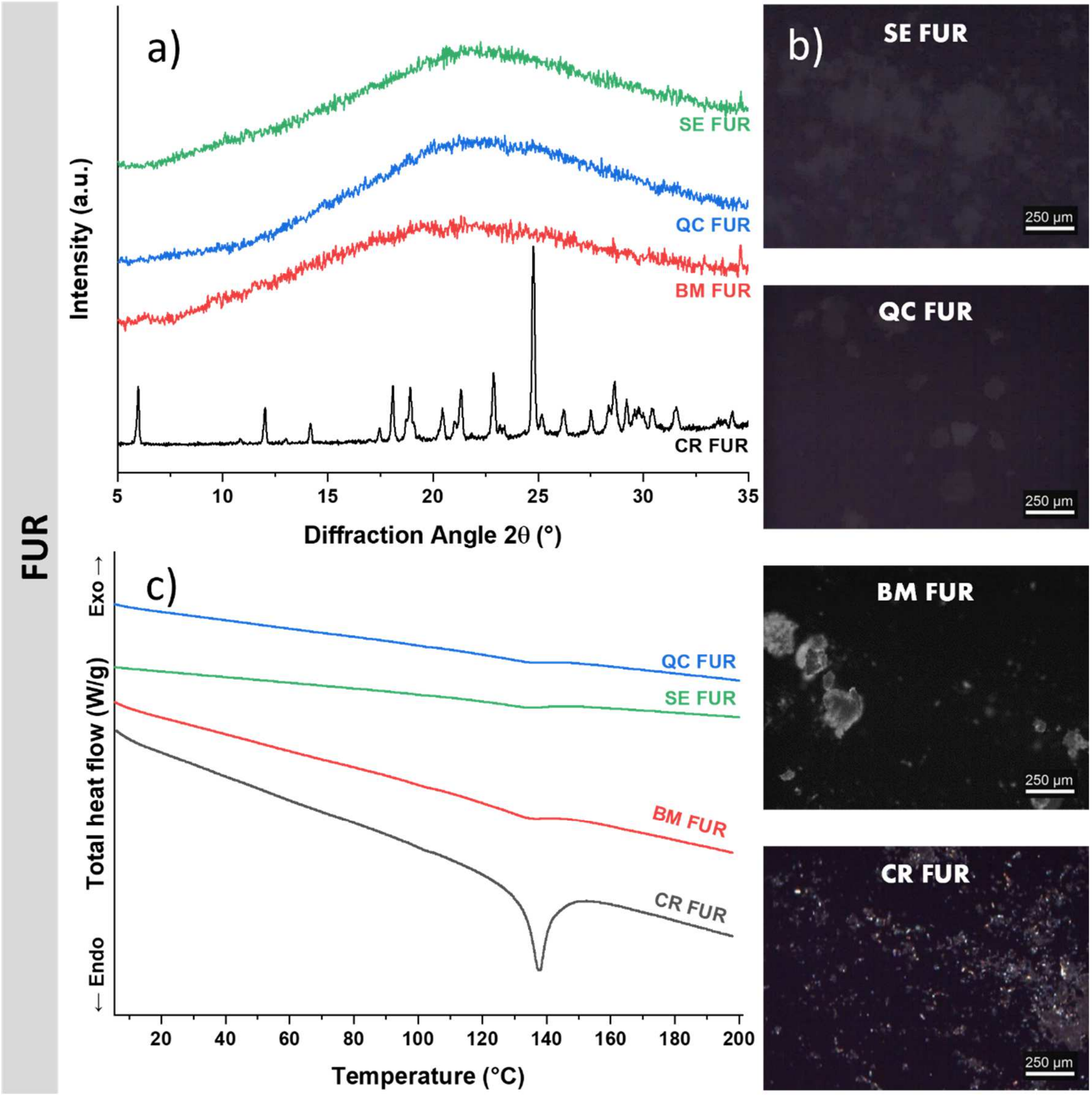

Figure S2. XRPD diffractograms (a), PLM micrographs (b), and DSC thermograms (c) of FUR. CR and samples after preparation by BM, QC, and SE are shown.

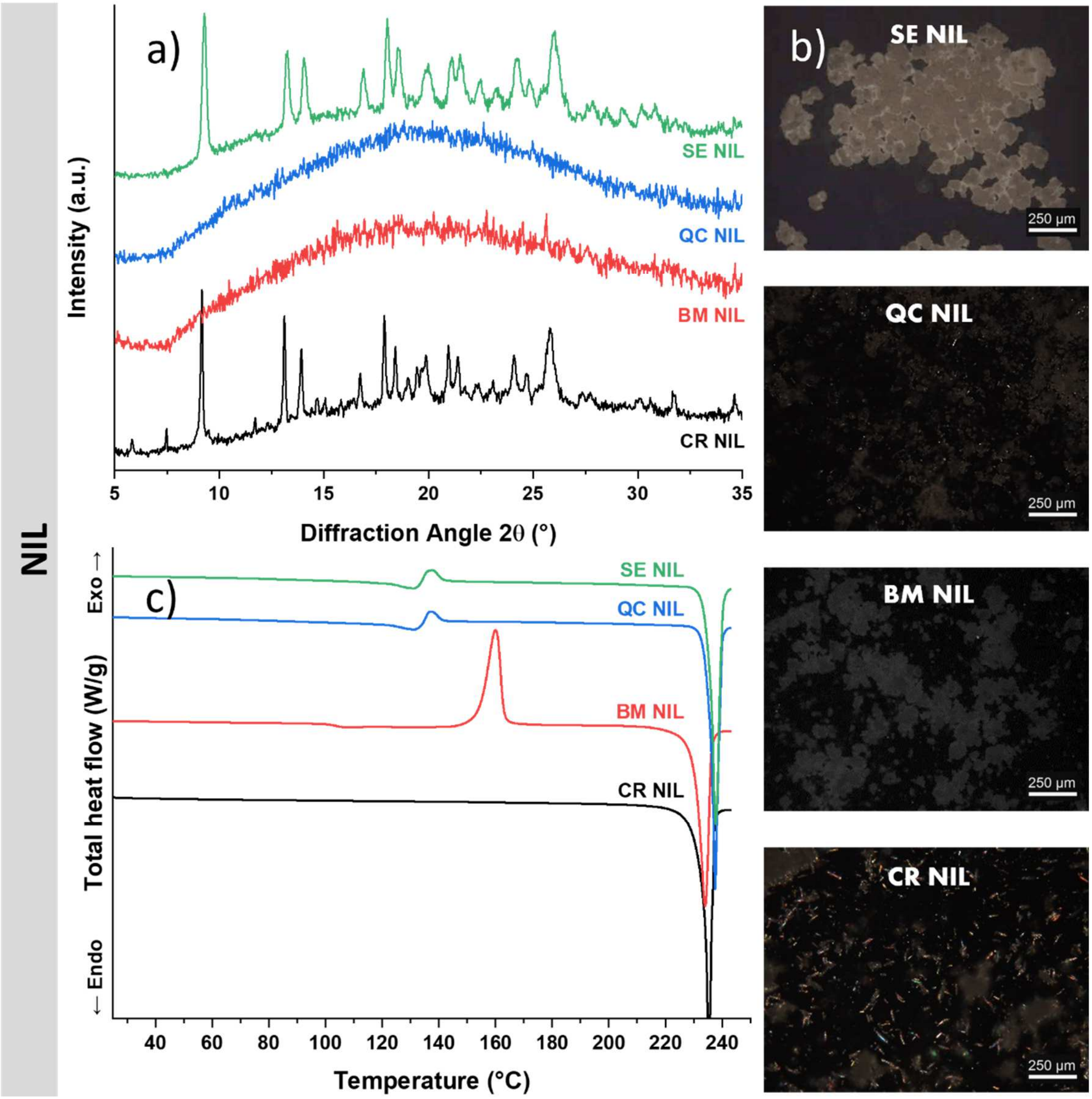

Figure S3. XRPD diffractograms (a), PLM micrographs (b), and DSC thermograms (c) of NIL. CR and samples after preparation by BM, QC, and SE are shown.

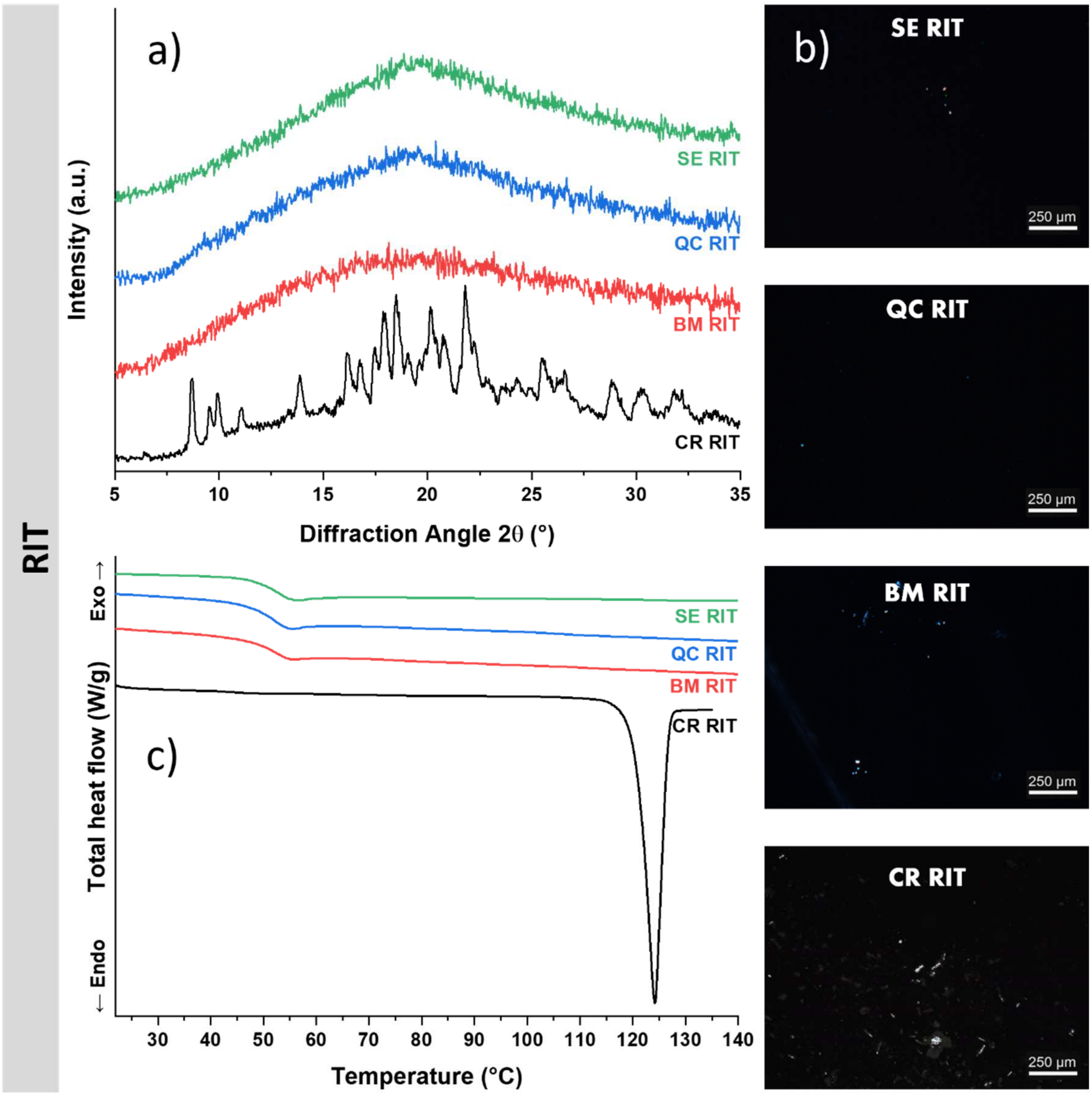

Figure S4. XRPD diffractograms (a), PLM micrographs (b), and DSC thermograms (c) of RIT. CR and samples after preparation by BM, QC, and SE are shown.

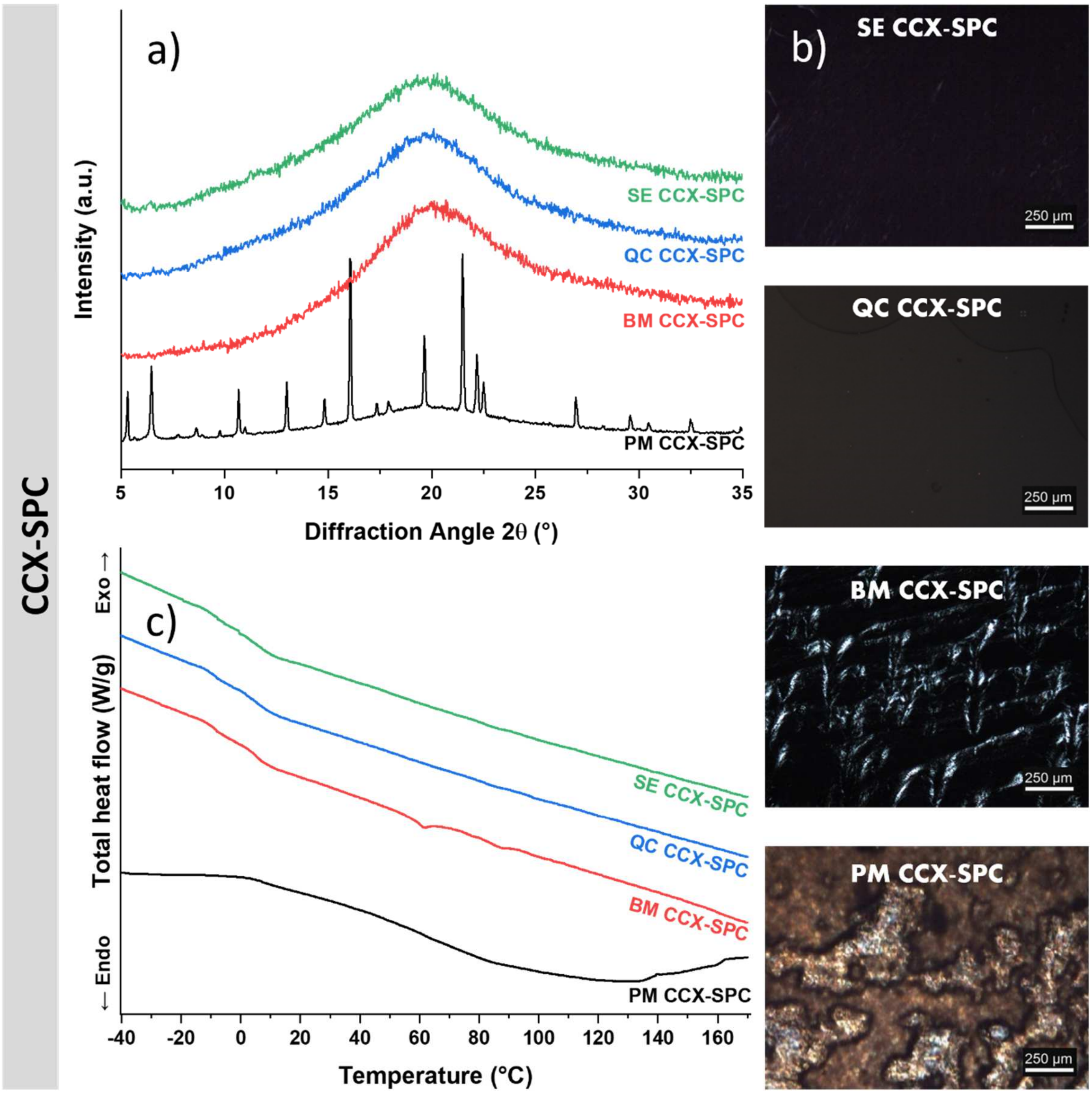

Figure S5. XRPD diffractograms (a), PLM micrographs (b), and DSC thermograms (c) of CCX-SPC at equimolar ratio. PM and samples after preparation by BM, QC, and SE are shown.

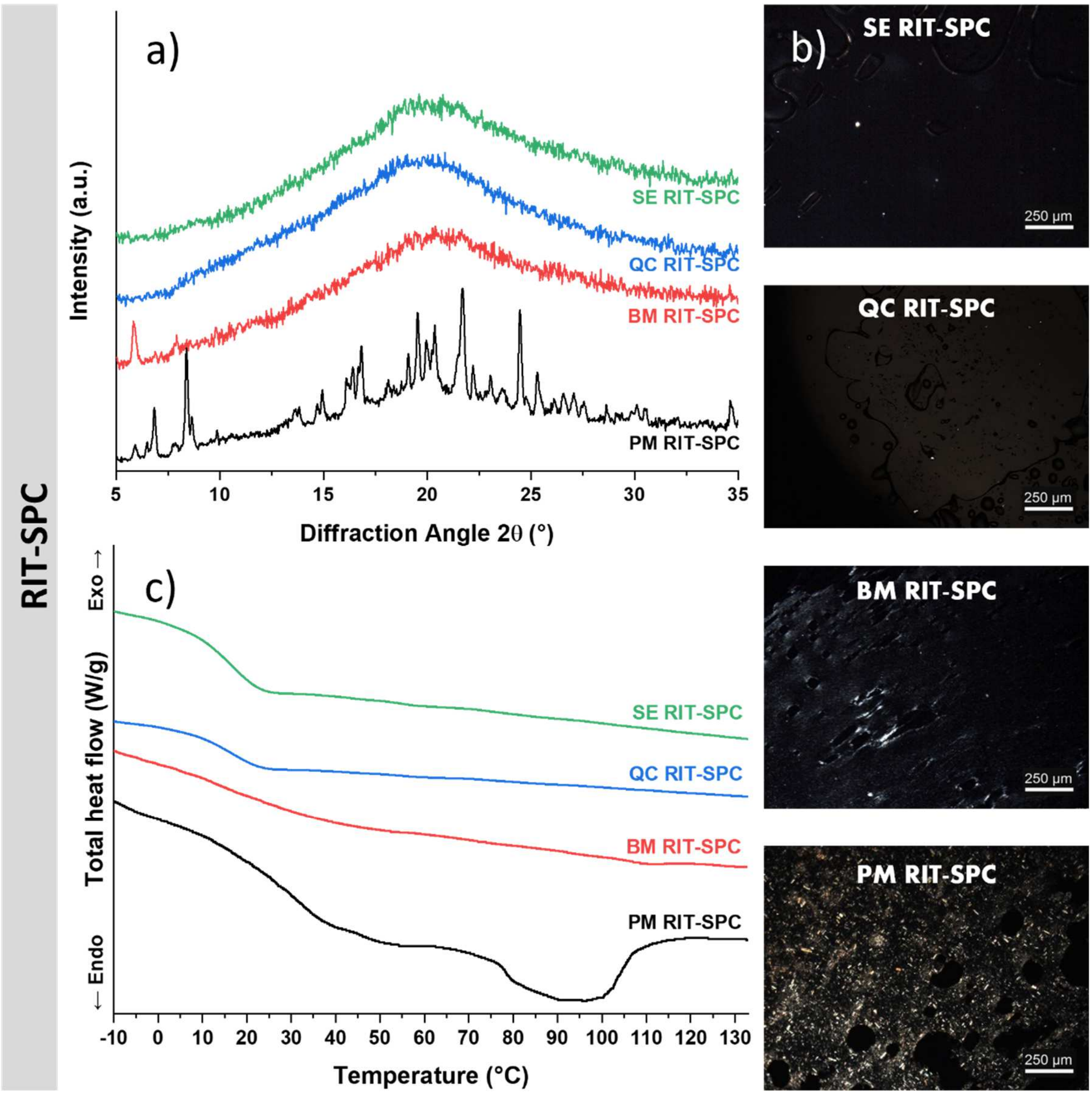

Figure S6. XRPD diffractograms (a), PLM micrographs (b), and DSC thermograms (c) of RIT-SPC at equimolar ratio. PM and samples after preparation by BM, QC, and SE are shown.

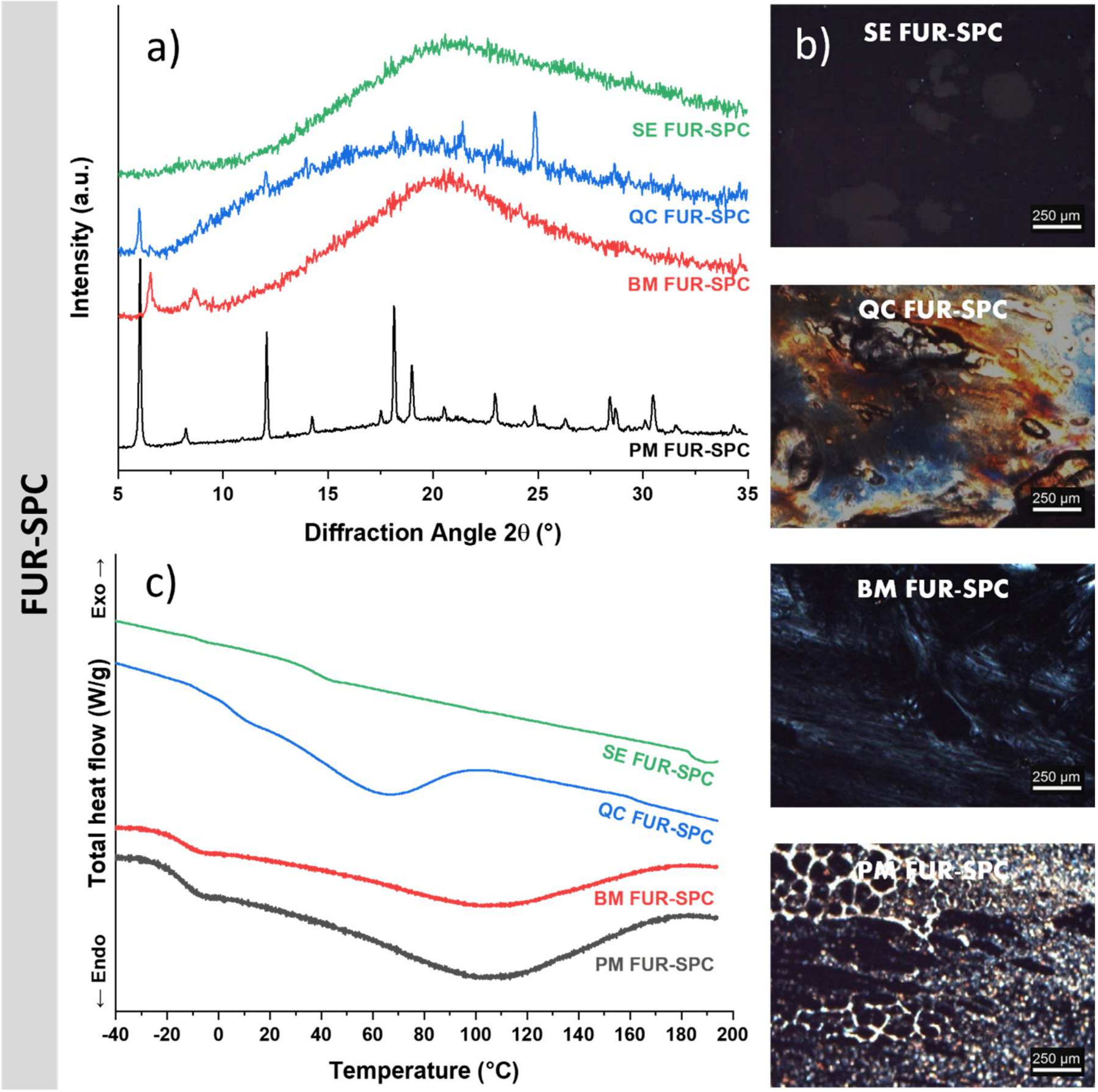

Figure S7. XRPD diffractograms (a), PLM micrographs (b), and DSC thermograms (c) of FUR-SPC at equimolar ratio. PM and samples after preparation by BM, QC, and SE are shown.

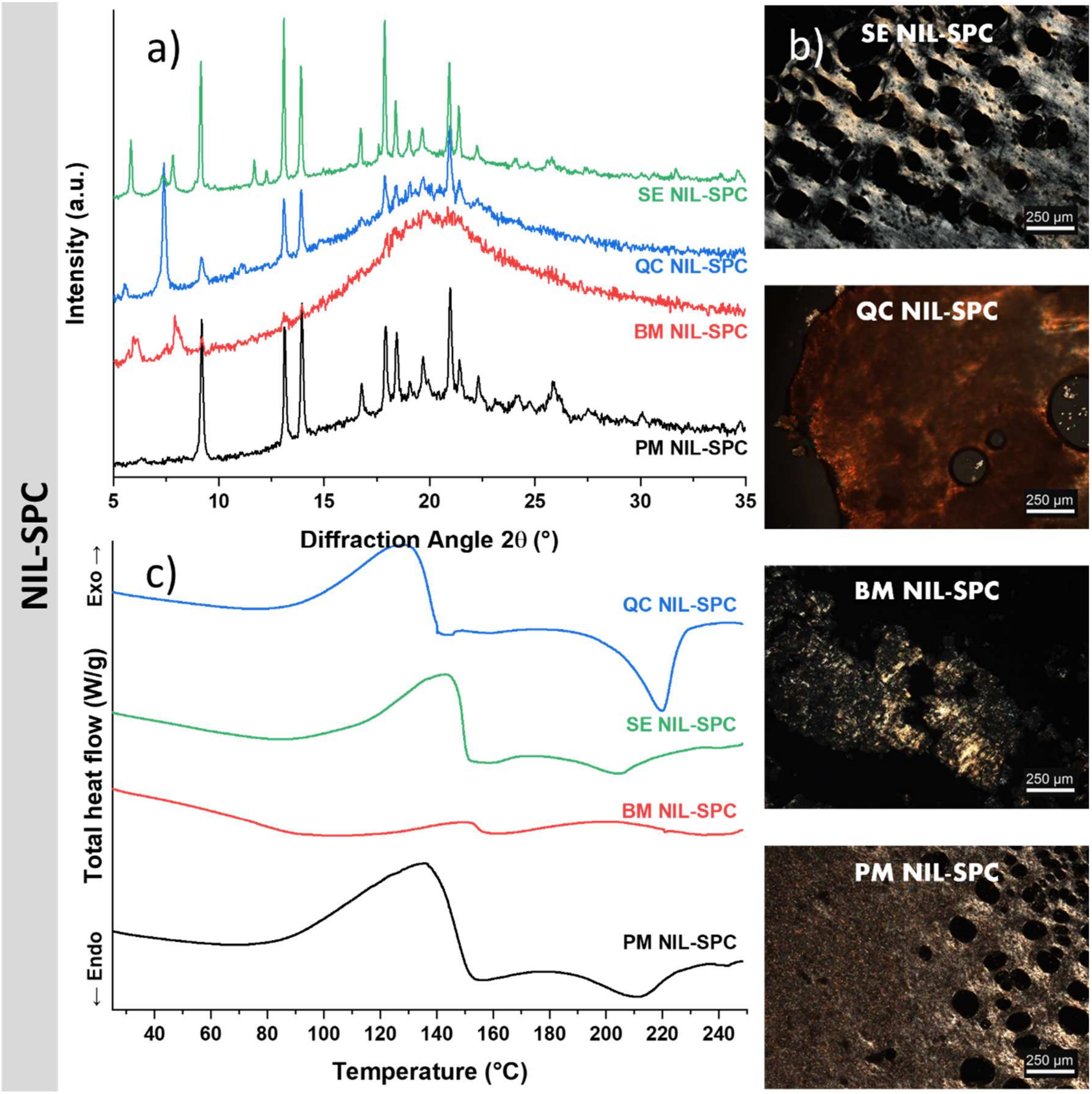

Figure S8. XRPD diffractograms (a), PLM micrographs (b), and DSC thermograms (c) of NIL-SPC at equimolar ratio. PM and samples after preparation by BM, QC, and SE are shown.

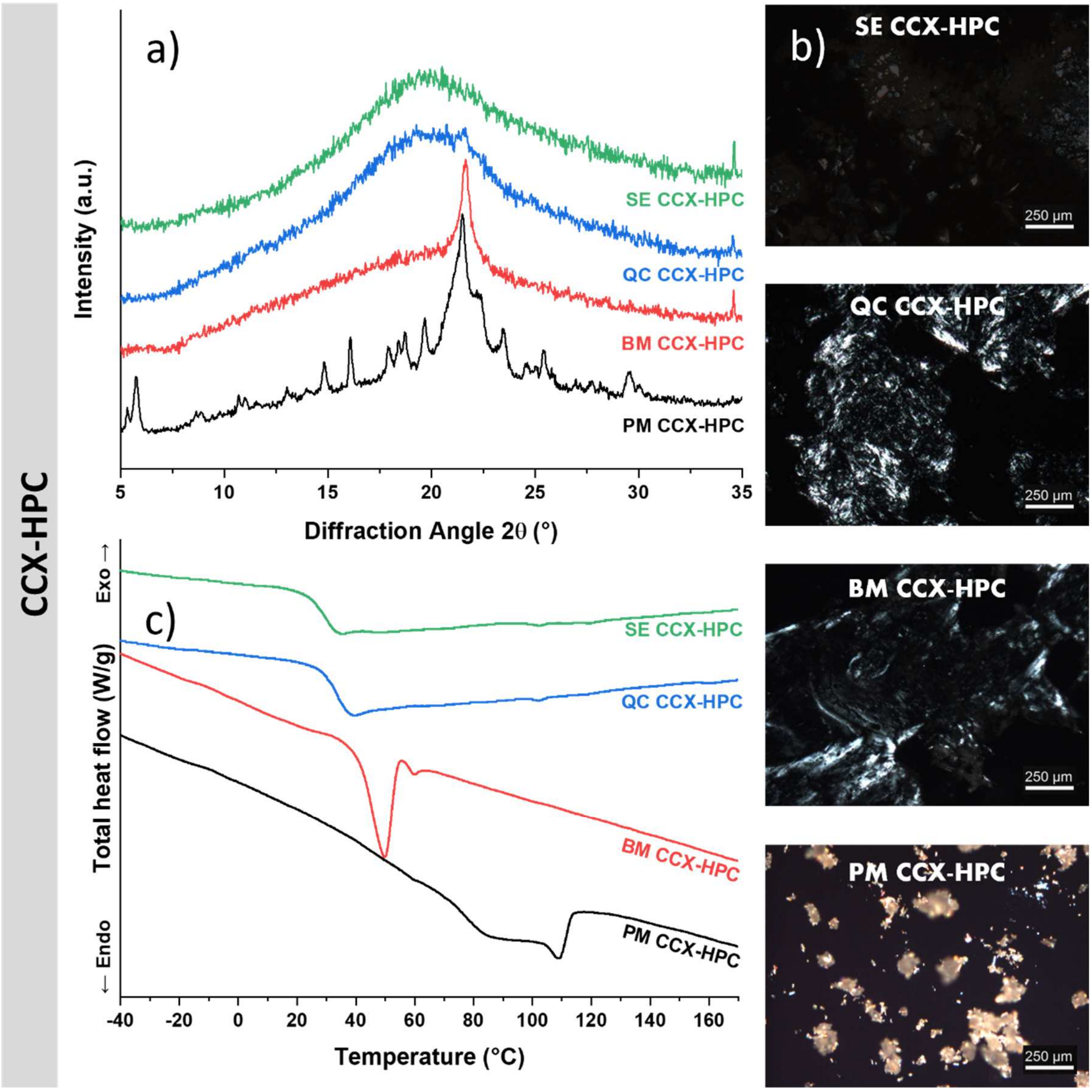

Figure S9. XRPD diffractograms (a), PLM micrographs (b), and DSC thermograms (c) of CCX-HPC at equimolar ratio. PM and samples after preparation by BM, QC, and SE are shown.

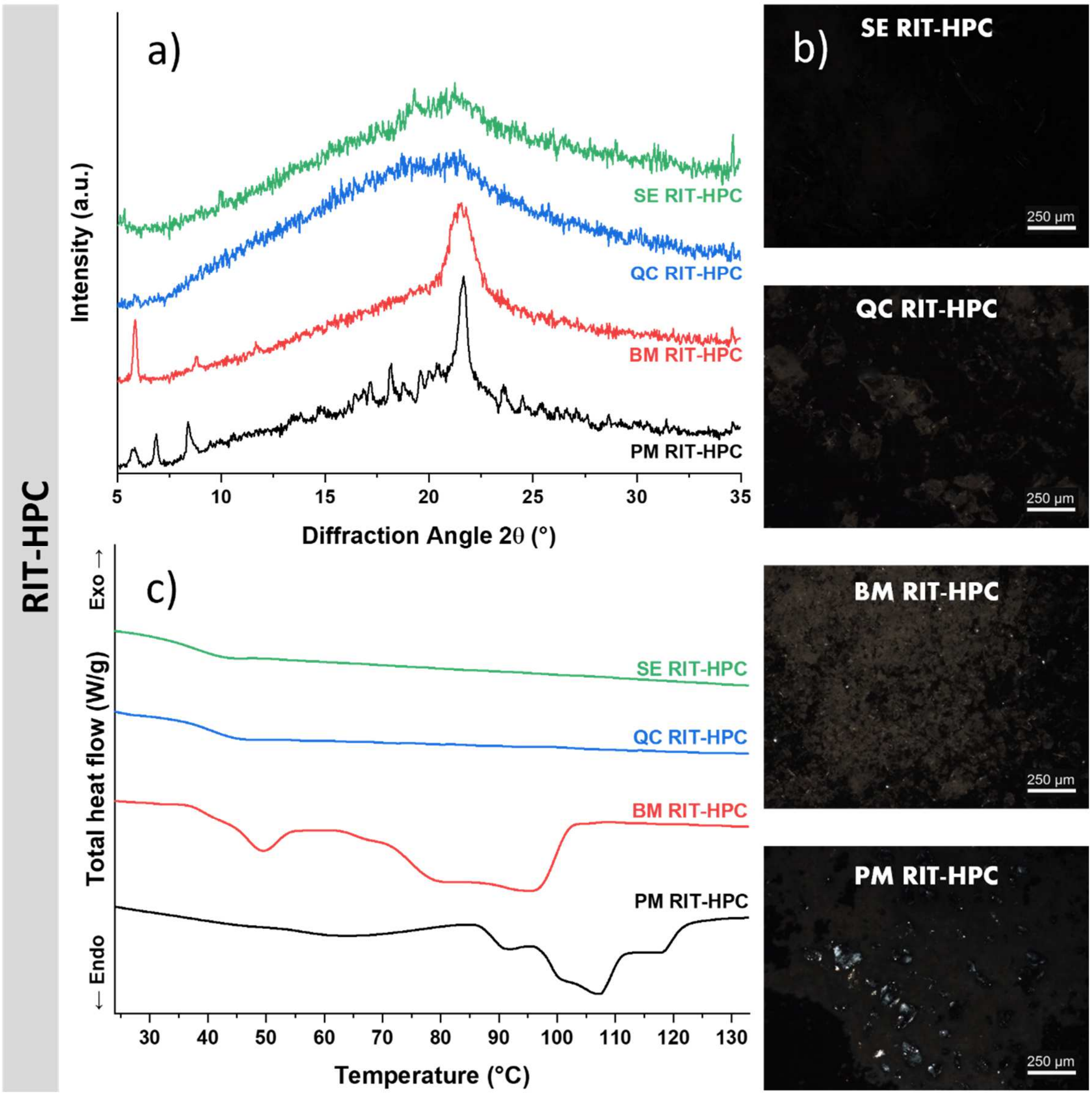

Figure S10. XRPD diffractograms (a), PLM micrographs (b), and DSC thermograms (c) of RIT-HPC at equimolar ratio. PM and samples after preparation by BM, QC, and SE are shown.

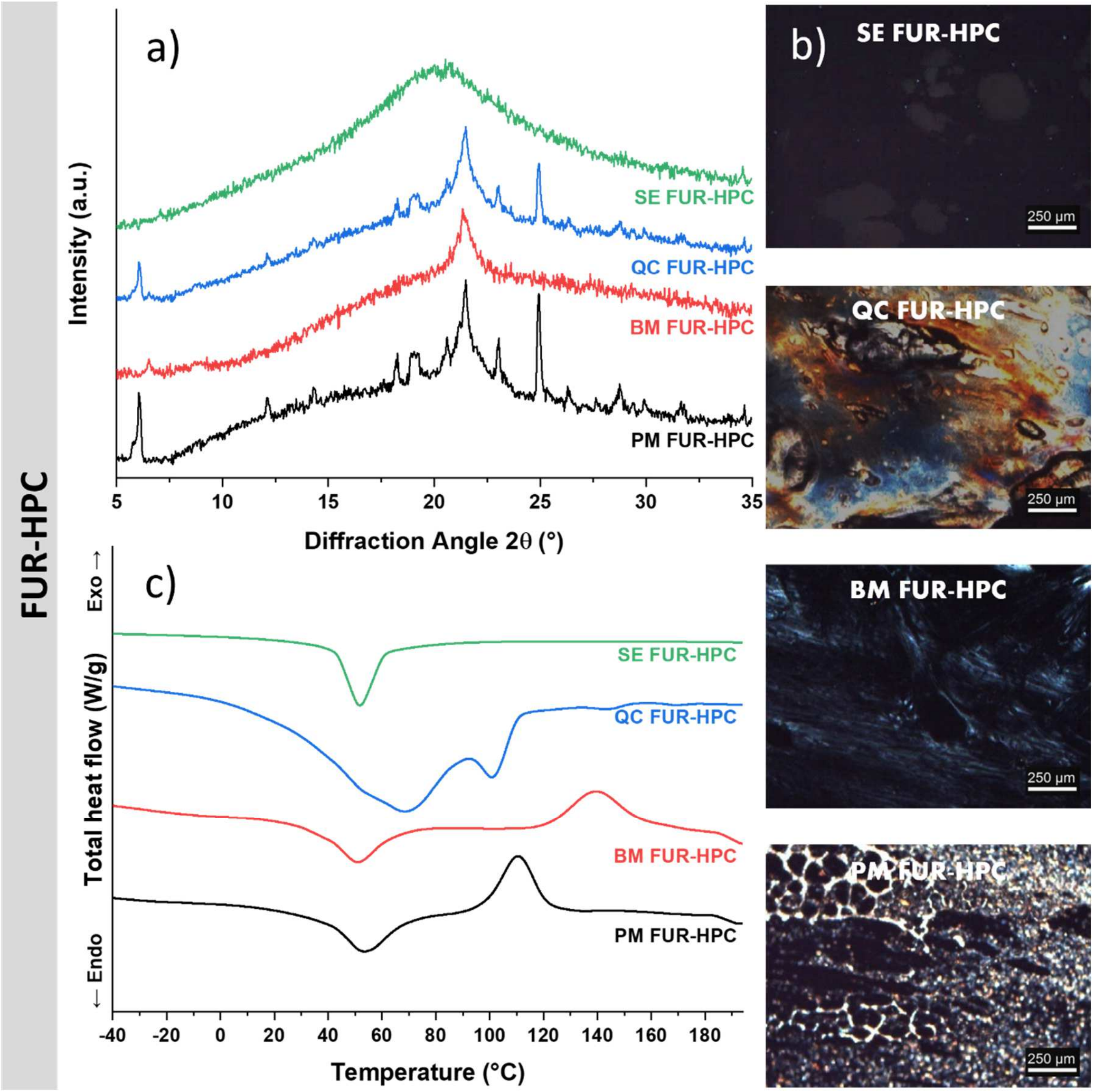

Figure S11. XRPD diffractograms (a), PLM micrographs (b), and DSC thermograms (c) of FUR-HPC at equimolar ratio. PM and samples after preparation by BM, QC, and SE are shown.

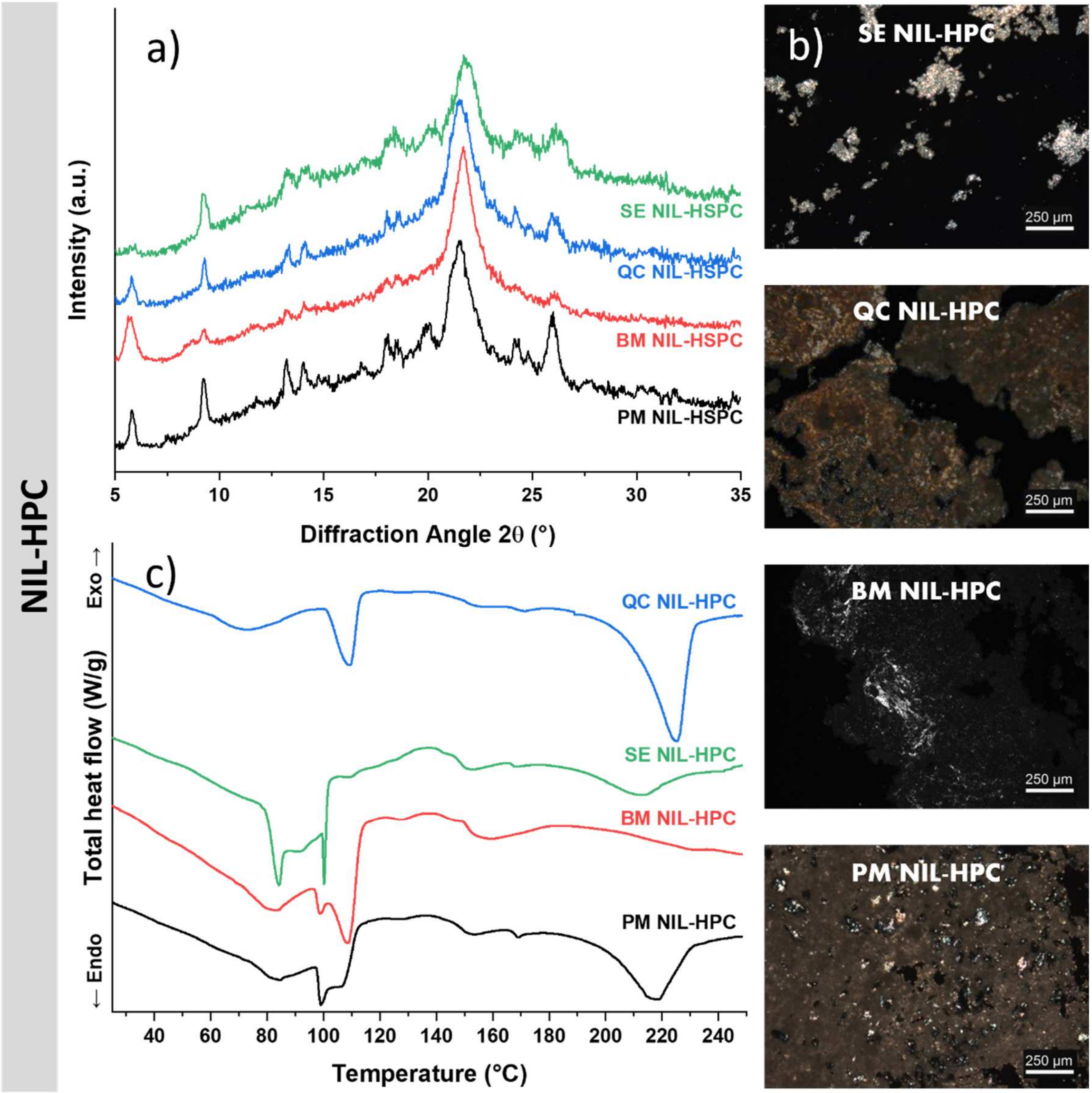

Figure S12. XRPD diffractograms (a), PLM micrographs (b), and DSC thermograms (c) of NIL-HPC at equimolar ratio. PM and samples after preparation by BM, QC, and SE are shown.

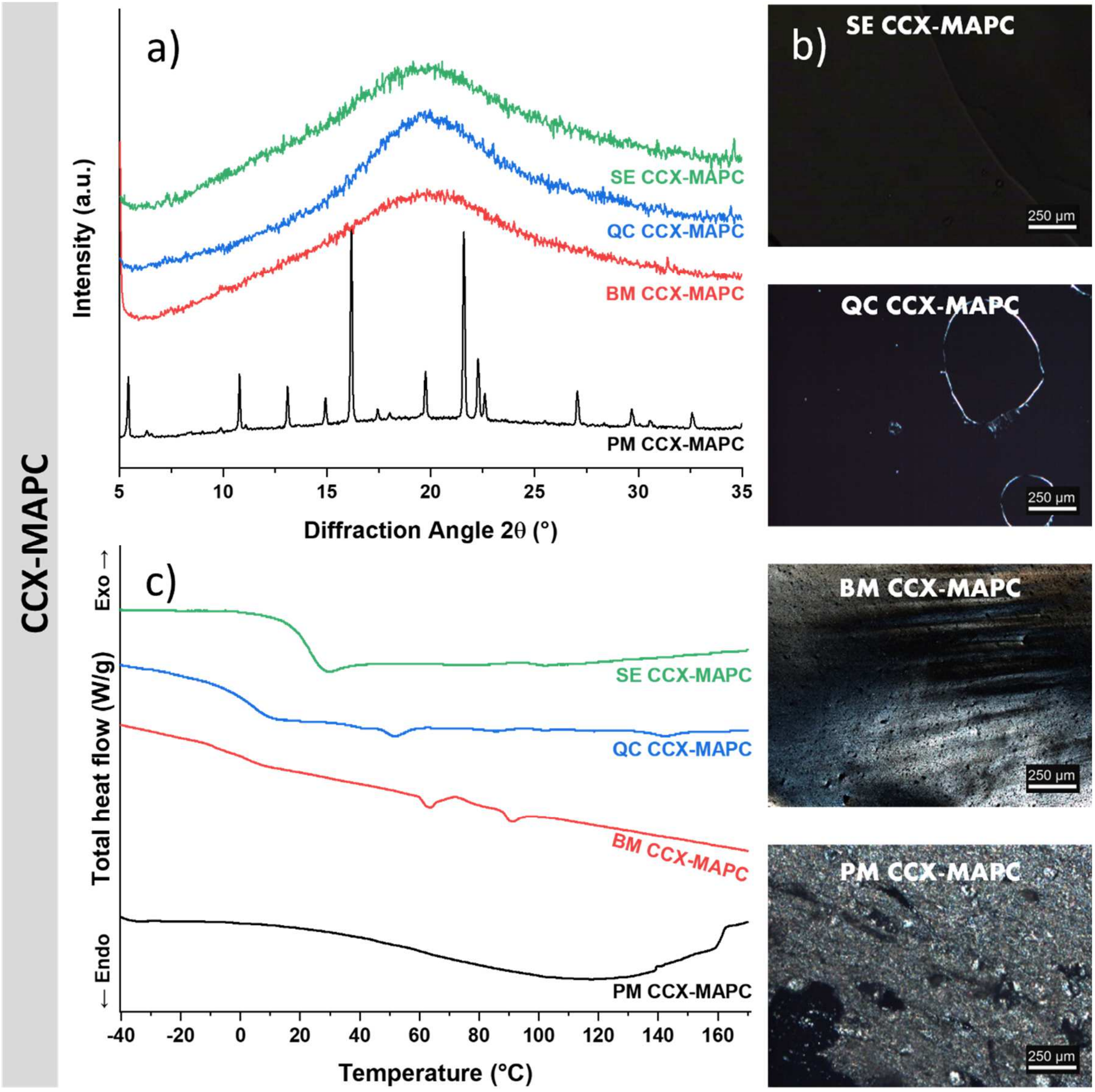

Figure S13. XRPD diffractograms (a), PLM micrographs (b), and DSC thermograms (c) of CCX-MAPC at equimolar ratio. PM and samples after preparation by BM, QC, and SE are shown.

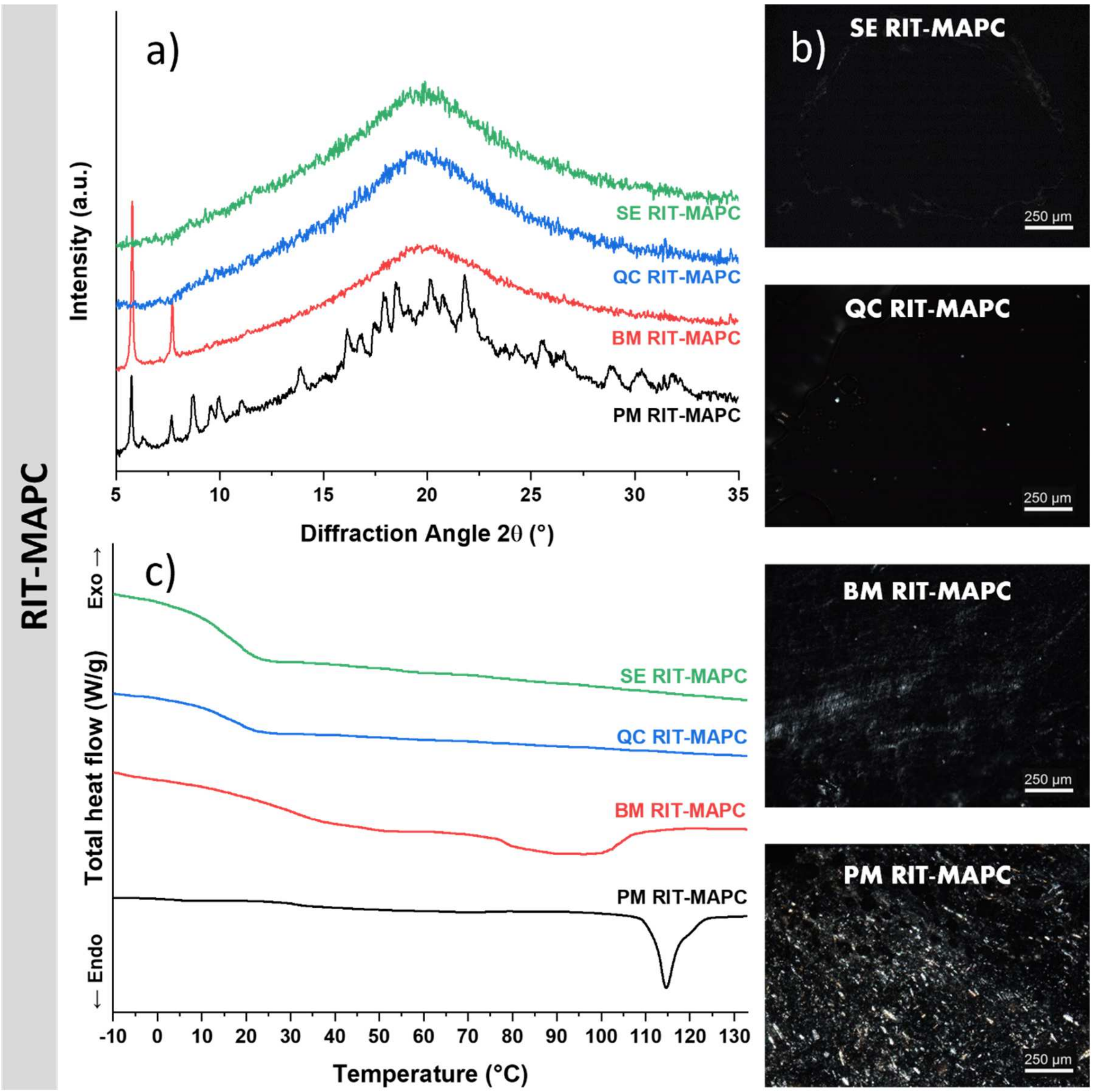

Figure S14. XRPD diffractograms (a), PLM micrographs (b), and DSC thermograms (c) of RIT-MAPC at equimolar ratio. PM and samples after preparation by BM, QC, and SE are shown.

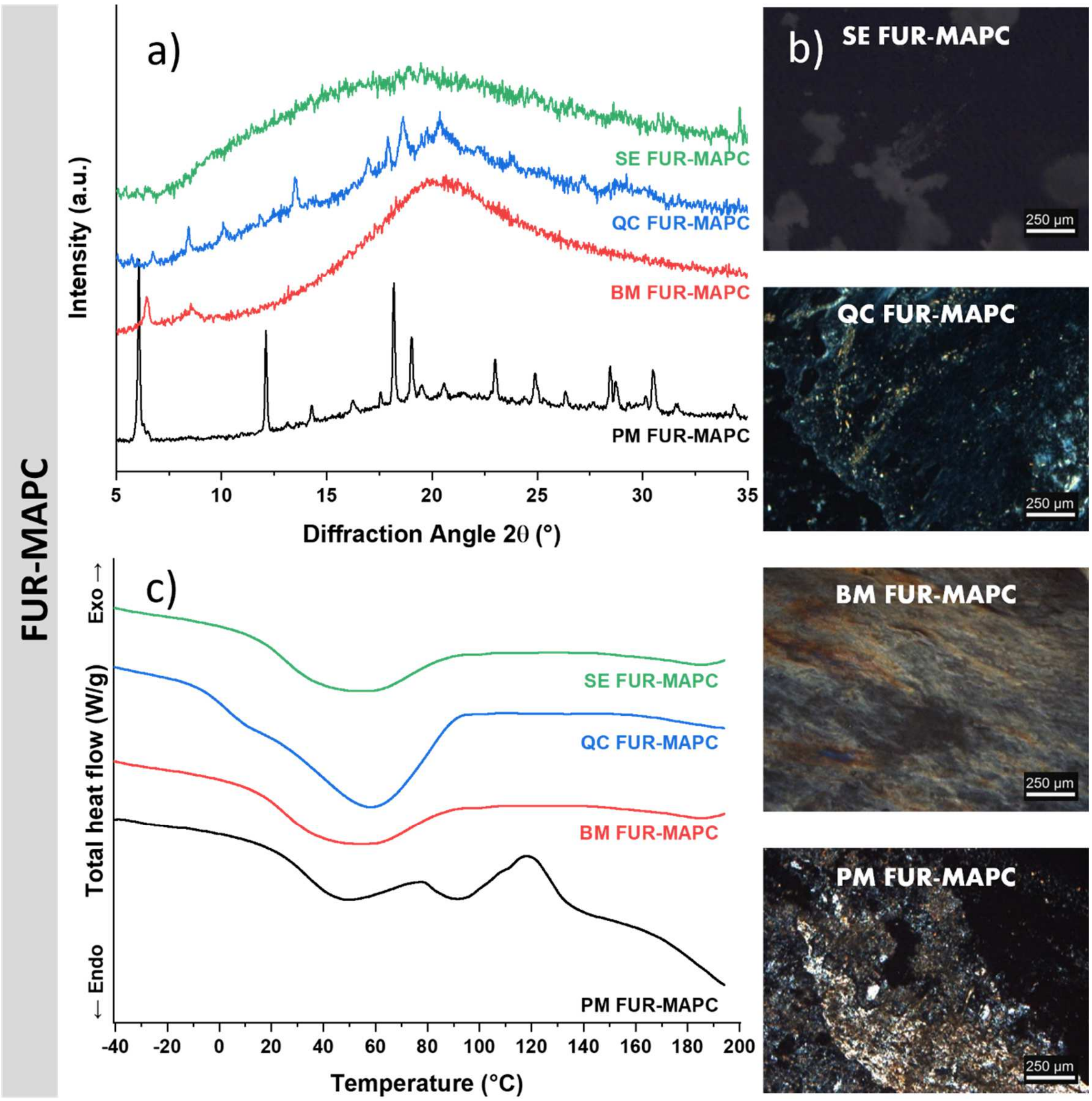

Figure S15. XRPD diffractograms (a), PLM micrographs (b), and DSC thermograms (c) of FUR-MAPC at equimolar ratio. PM and samples after preparation by BM, QC, and SE are shown.

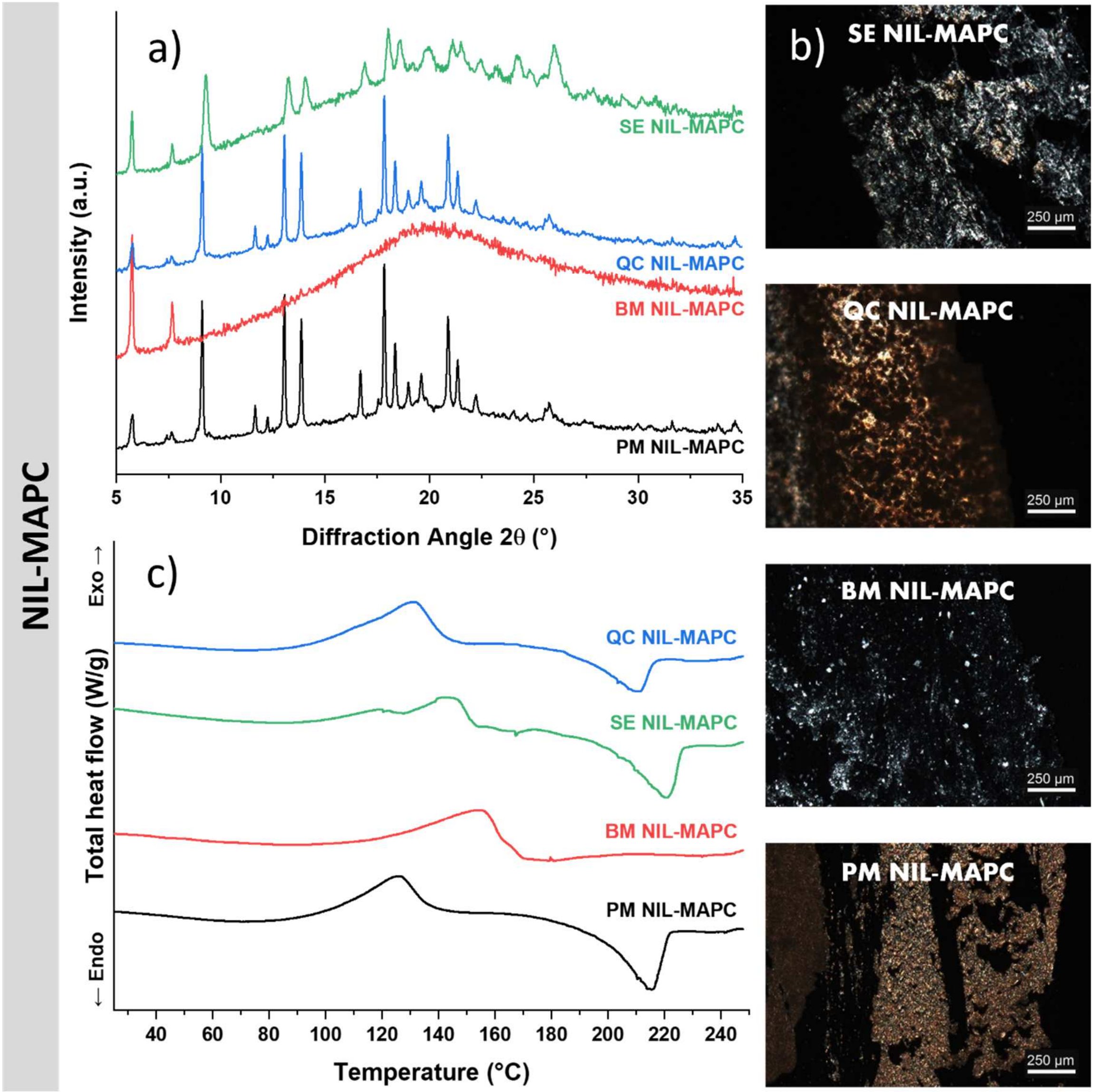

Figure S16. XRPD diffractograms (a), PLM micrographs (b), and DSC thermograms (c) of NIL-MAPC at equimolar ratio. PM and samples after preparation by BM, QC, and SE are shown.
